# Supplementary material for: Distinct neural representations for prosocial and self-benefiting effort
Source: Curr Biol. 2022 Oct 10;32(19):4172–4185.e7. doi: 10.1016/j.cub.2022.08.010 (PMC9616728; doi:10.1016/j.cub.2022.08.010)
Supplement: Document S1. Figures S1–S6 and Tables S1–S7 [file mmc1.pdf]

Current Biology, Volume *32*

# Supplemental Information

# Distinct neural representations

# for prosocial and self-benefiting effort

**Patricia L. Lockwood, Marco K. Wittmann, Hamed Nili, Mona Matsumoto-Ryan, Ayat Abdurahman, Jo Cutler, Masud Husain, and Matthew A.J. Apps**

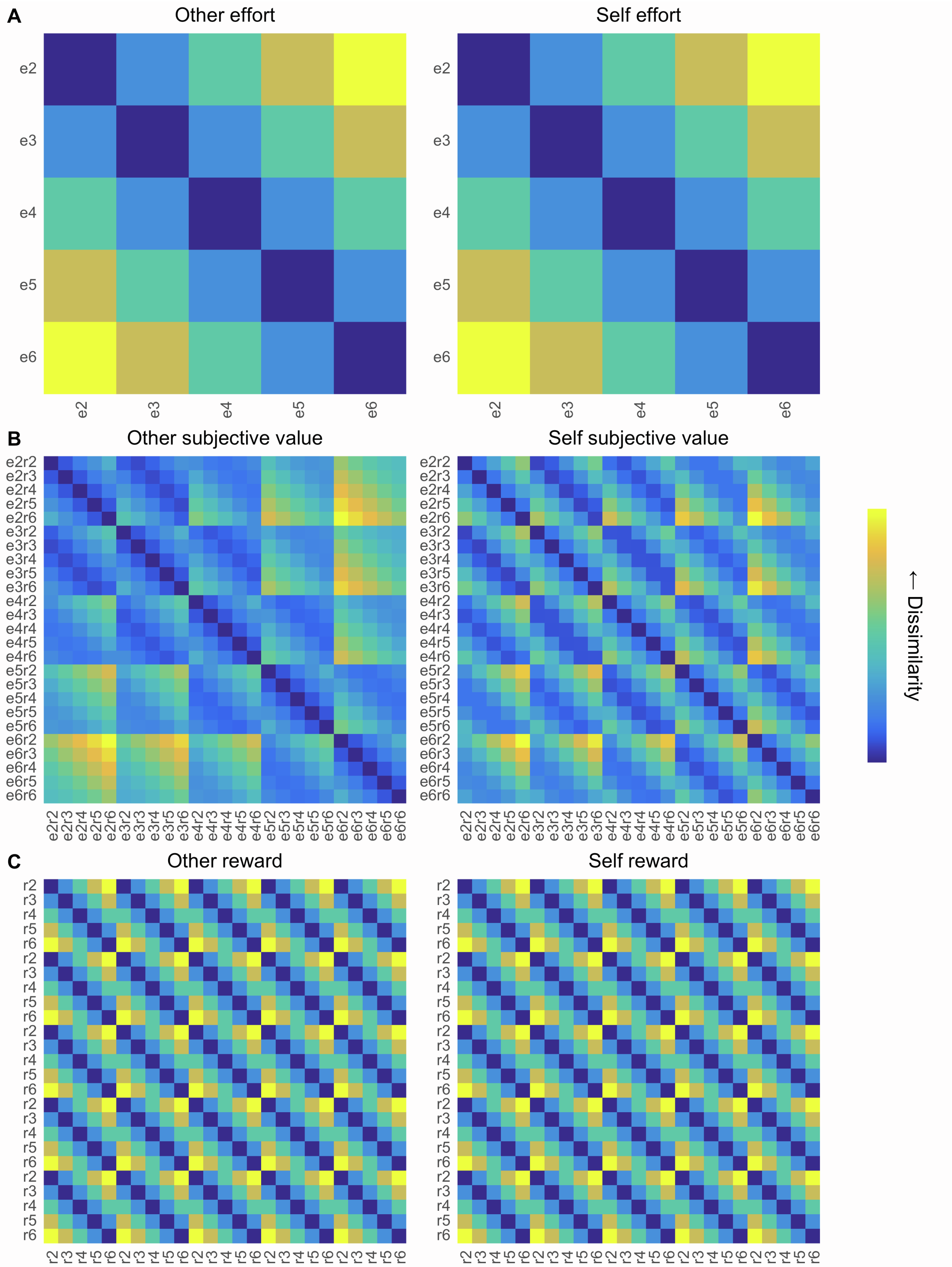


**Figure S1. Model RDMs for effort, subjective value and reward. Related to Figure 1.** Six 25 x 25 (5 effort and 5 reward levels) model representational dissimilarity matrices (RDMs) were constructed at the offer stage to analyse activity during effort-based decisions separately for the self and other conditions. These coded for different task features in multivariate space. **(A)** For the effort model RDMs, this was the Euclidean distance between effort levels on offer. **(B)** For the subjective value RDMs, this was the Euclidean distance between subjective values of offers based on the winning computational model with separate discount parameters (*K*) for self and other trials. Each participant's individual *K*

parameter was used. **(C)** For the reward RDMs this was the Euclidean distance between reward levels on offer. Yellow colours show conditions are more dissimilar whereas dark blue colours show conditions are more similar in terms of the Euclidean distance between conditions.

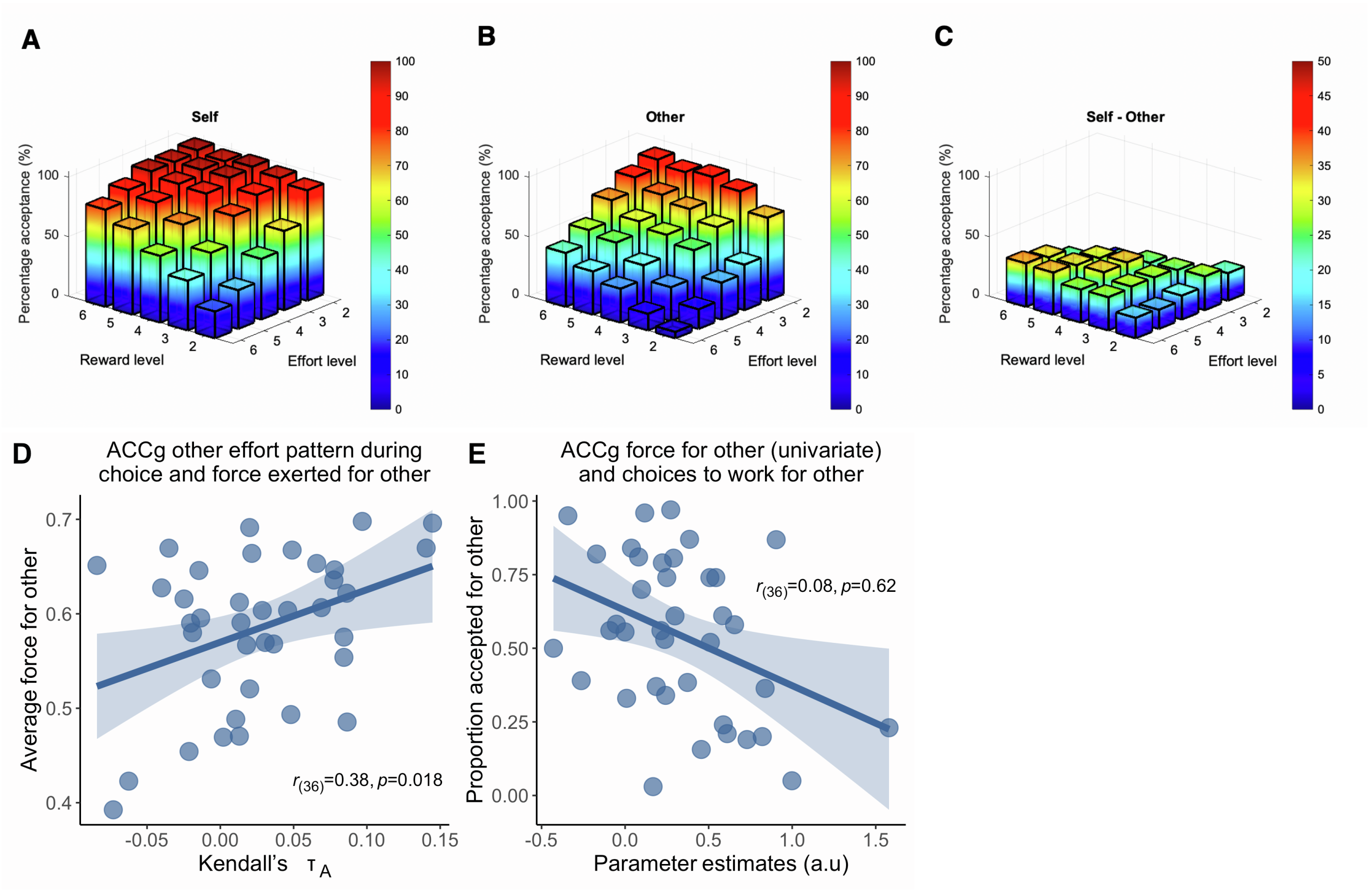


**Figure S2. Supplemental behavioural analysis of choice and force. Related to Figure 2.** 3D plots showing percentage acceptance of work over rest offers on self and other trials and the difference between them**. (A)** Percentage acceptance on self trials as a function of reward and effort level. **(B)** Percentage acceptance on other trials as a function of reward and effort level. **(C)** Difference in percentage acceptance between self and other trials. **(D)** ACCg representations of effort for another person during the choice phase, taken from the RSA analysis, positively correlate with amount of force subsequently exerted for other (Pearson's $r_{(36)}$=0.38, $p$=0.018). **(E)** Univariate responses in ACCg to force required for other negatively correlate with proportion of choices to benefit other (Pearson's $r_{(36)}$=-0.38, $p$=0.018).

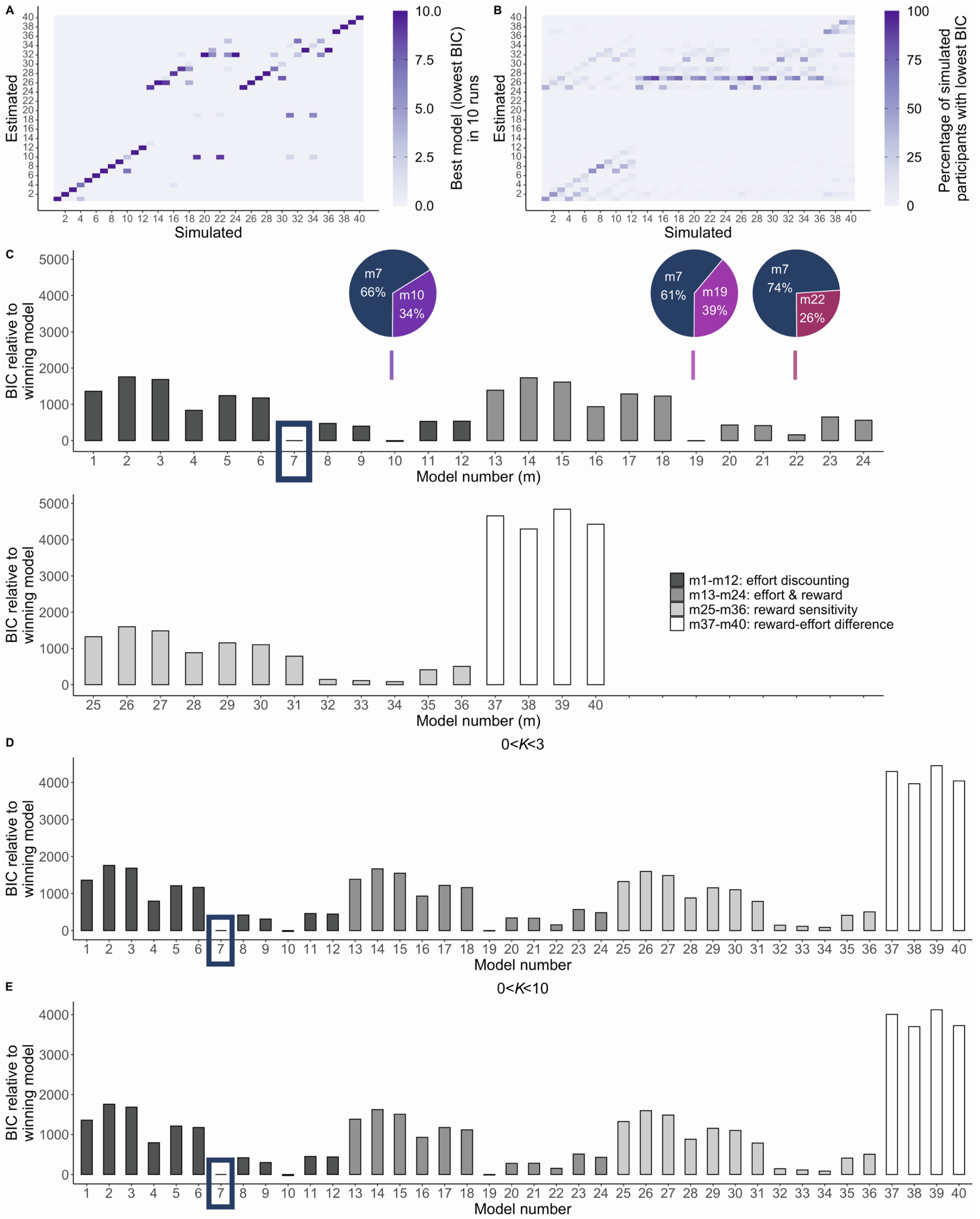


**Figure S3. Model identifiability and model comparison with all models and different *K* bounds. Related to Figure 2. (A)** Data simulated from each of the 40 models (see section ‘Computational Modelling’ below for details) for 100 participants. The model comparison procedure identifies the model that simulated the data, demonstrated by the strong diagonal, for models 1-12, which include an effort discounting and an inverse temperature parameter. However, models 13-24 (additional reward sensitivity parameter), models 25-36 (only a reward sensitivity parameter, no effort discounting parameter) and models 37-40 (linear models only, parameter scaling difference between effort and reward) show poor identifiability. **(B)** We repeated the simulations and fittings ten times and quantified the winning model as

with the modelling of participants' data as the model with the lowest Bayesian Information Criterion (BIC), summing the number of times that model won across the ten runs. We also calculated the percentage of simulated participants for which each model had the best fit to the data and averaged this over the ten runs. **(C)** Model comparison of all 40 models shows that even those models with comparable BICs explain behaviour in fewer participants than model 7. **(D)** We also tested empirically whether the estimated *K's* were sensitive to the bounds we placed on them. Increasing the maximum *K* value to 3 did not change the winning model (Model 7). **(E)** Repeating the model fitting with the upper *K* bound as 10 also gave identical results.

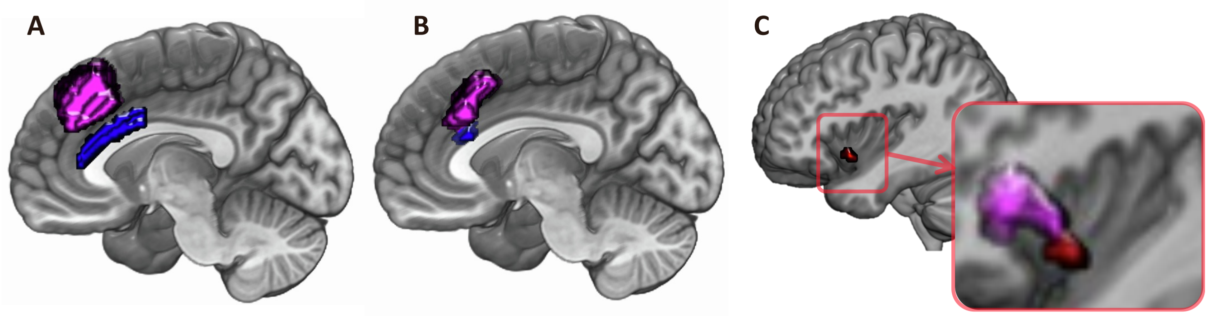


**Figure S4. Distinct portions of the cingulate cortex involved in domain general and domain specific computations. Related to Figure 4, Table S6 and S7. (A)** anatomical regions of interest in ACCg (blue) and dACC/dmPFC (pink) overlaid on an anatomical scans of the medial surface. Notably, the two areas of the cingulate cortex are distinct. Whereas the ACCg signalled representations of effort for others only, the dACC/dmPFC signalled patterns of subjective value for both self and other. **(B)** univariate analyses show that activation in the ACCg (blue) for force exerted for others only is distinct from a separate areas of the anterior cingulate cortex in the dACC/dmPFC (pink) that negatively tracked trial-by-trial subjective value for both self and other. **(C)** univariate analysis show that activation in the ventral anterior insula (vAI) that responds more strongly on self than other trials does not overlap with the domain general portion of anterior insula (pink) that tracks subjective value in a conjunction analysis for both self and other.

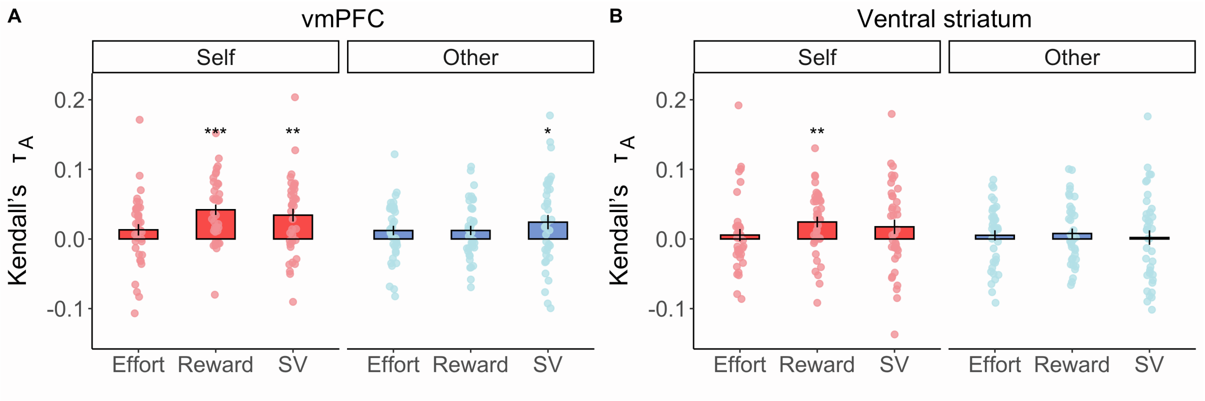


**Figure S5. vmPFC and VS multivariate patterns of effort, reward and subjective value. Related to Table S6 and STAR Methods.** We conducted additional exploratory analysis with two ROIs in vmPFC and VS that have more recently been suggested to encode effort, reward and subjective value. We found vmPFC coded reward and subjective value for self but only subjective value for other. In contrast ventral striatum only encoded reward for self. Reward was represented more strongly than effort in vmPFC for self but this was not the case for other (Table S6).

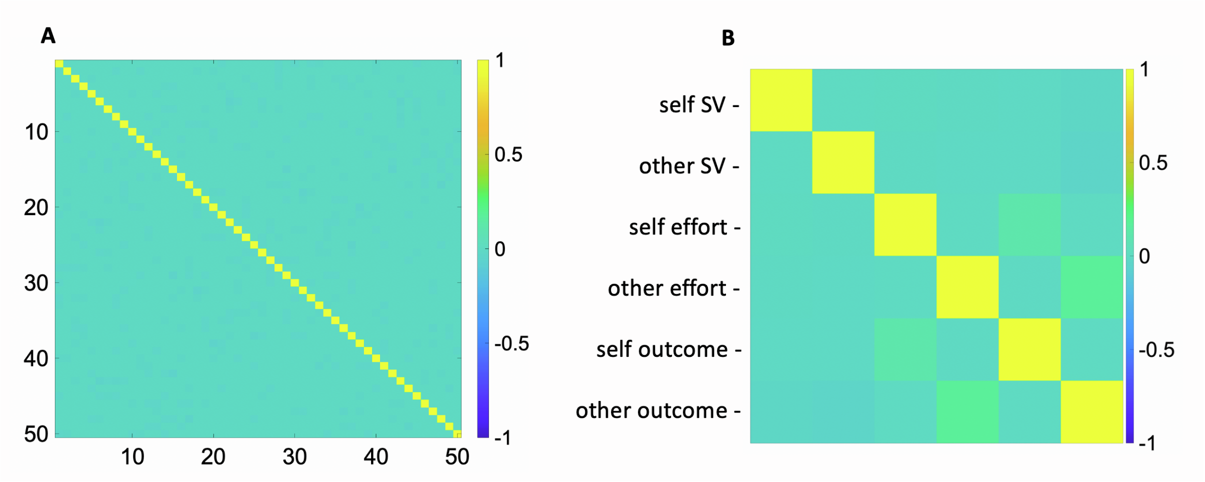


**Figure S6. Multivariate and univariate GLMs were decorrelated. Related to STAR Methods. (A)** Correlation between regressors in the Representational Similarity Analysis GLM. A 50x50 GLM of regressors modelled each effort/reward combination separately for self (columns 1-25) and other (columns 26-50) trials. Within this GLM all correlations were below r<l0.007l, indicating that conditions could be appropriately estimated with independence from one another. The GLM also modelled the onset of the force period and the onset of the outcome for each recipient (self and other). **(B)** Correlation between regressors in the univariate GLM. The correlations between choice (subjective value) and effort parametric regressors were below r<l0.01l, indicating that all conditions of interest (subjective value at the offer stage, effort required at the force stage) could be appropriately estimated with independence from one another. SV: subjective value.

**Table S1. Generalised linear mixed-effects model predicting choices. Related to Figure 2.**

| | OR | SE | CI low | CI up | Z | χ2 | df | *p* |
|---|---|---|---|---|---|---|---|---|
| (Intercept) | 6.98 | 2.52 | 3.44 | 14.15 | 5.39 | | | |
| Recipient (Self vs. Other) | 4.04 | 0.99 | 2.49 | 6.54 | 5.66 | 23.05 | 1 | 0.001 |
| Effort | 0.13 | 0.02 | 0.09 | 0.17 | -12.38 | 64.24 | 1 | 0.001 |
| Reward | 6.10 | 1.18 | 4.18 | 8.90 | 9.38 | 46.05 | 1 | 0.001 |
| Recipient (Self vs. Other) * Effort | 1.20 | 0.09 | 1.03 | 1.40 | 2.40 | 6.53 | 1 | 0.011 |
| Recipient (Self vs. Other) * Reward | 1.31 | 0.11 | 1.11 | 1.55 | 3.20 | 9.51 | 1 | 0.003 |
| Effort * Reward | 1.18 | 0.11 | 0.99 | 1.42 | 1.83 | 3.38 | 1 | 0.088 |
| Recipient (Self vs. Other) * Effort * Reward | 0.97 | 0.05 | 0.87 | 1.09 | -0.47 | 0.19 | 1 | 0.67 |

Note. OR: odds ratio, SE: standard error, CI: 95% confidence interval for odds ratio, low: lower CI, up: upper CI, *p* values are from type 2 tests of fixed effects using parametric bootstrapping (see Methods).

**Table S2. Linear mixed-effects model predicting normalised force. Related to Figure 3.**

| | $\chi 2$ | df | *p* |
|---|---|---|---|
| Recipient (Self vs. Other) | 7.09 | 1 | 0.013 |
| Effort | 409.15 | 4 | 0.001 |
| Reward | 65.66 | 4 | 0.001 |
| Recipient (Self vs. Other) * Effort | 5.36 | 4 | 0.24 |
| Recipient (Self vs. Other) * Reward | 13.21 | 4 | 0.014 |
| Effort * Reward | 49.88 | 16 | 0.001 |
| Recipient (Self vs. Other) * Effort * Reward | 42.03 | 16 | 0.002 |

Note. df: degrees of freedom, *p* values are from type 2 tests of fixed effects using parametric bootstrapping (see Methods).

**Table S3. Kendall's $\tau_A$ correlations between brain RDMs and model RDMs for additional ROIs. Related to STAR Methods.**

| | | Mean | SE | *p* | FDR *p* |
|---|---|---|---|---|---|
| Other effort RDM | vmPFC | 0.01 | 0.01 | 0.03 | 0.06 |
| | VS | 0.01 | 0.01 | 0.24 | 0.29 |
| Self effort RDM | vmPFC | 0.01 | 0.01 | 0.03 | 0.06 |
| | VS | 0.01 | 0.01 | 0.61 | 0.61 |
| Other reward RDM | vmPFC | 0.01 | 0.01 | 0.05 | 0.09 |
| | VS | 0.01 | 0.01 | 0.24 | 0.29 |
| Self reward RDM | vmPFC | 0.04 | 0.01 | <0.001 | <0.001 |
| | VS | 0.02 | 0.01 | 0.00 | 0.01 |
| Other subjective value RDM | vmPFC | 0.02 | 0.01 | 0.02 | 0.05 |
| | VS | 0.00 | 0.01 | 0.49 | 0.54 |
| Self subjective value RDM | vmPFC | 0.03 | 0.01 | <0.001 | 0.00 |
| | VS | 0.02 | 0.01 | 0.06 | 0.09 |

Note. RDM: representational dissimilarity matrix, Mean: Kendall's $\tau_A$ correlations between brain RDMs and model RDMs, SE: standard error of the mean, FDR *p*: false-discovery rate corrected *p* value across 12 comparisons.

**Table S4. Comparing RDMs between effort and reward for each recipient. Related to STAR Methods.**

| | | Z | *r* | CI low | CI up | *p* |
|---|---|---|---|---|---|---|
| Reward vs. effort self RDM | vmPFC | -2.28 | 0.37 | 0.06 | 0.62 | 0.02 |
| | VS | -1.49 | 0.24 | 0.02 | 0.54 | 0.14 |
| Reward vs. effort other RDM | vmPFC | -0.50 | 0.08 | 0.01 | 0.41 | 0.62 |
| | VS | -0.17 | 0.03 | 0.00 | 0.37 | 0.86 |

Note. RDM: representational dissimilarity matrix, *r*: standardised effect size, CI: 95% confidence interval for odds ratio, low: lower CI, up: upper CI, values are from Wilcoxon two-sided rank tests comparing effort and reward.

**Table S5. Kendall's $\tau_A$ correlations between brain RDMs and model RDMs. Related to Figure 4.**

| | | Mean | SE | *p* | FDR *p* |
|---|---|---|---|---|---|
| Other effort RDM | ACCg | 0.026 | 0.009 | 0.005 | 0.009 |
| | AI | 0.021 | 0.008 | 0.006 | 0.009 |
| | dACC/dmPFC | 0.029 | 0.008 | 0.001 | 0.003 |
| | TPJ | 0.033 | 0.010 | 0.001 | 0.003 |
| Self effort RDM | ACCg | 0.002 | 0.009 | 0.610 | 0.610 |
| | AI | 0.008 | 0.009 | 0.400 | 0.420 |
| | dACC/dmPFC | 0.016 | 0.012 | 0.160 | 0.170 |
| | TPJ | 0.024 | 0.013 | 0.026 | 0.032 |
| Other reward RDM | ACCg | 0.009 | 0.007 | 0.16 | 0.170 |
| | AI | 0.020 | 0.008 | 0.006 | 0.009 |
| | dACC/dmPFC | 0.025 | 0.007 | 0.001 | 0.003 |
| | TPJ | 0.016 | 0.007 | 0.027 | 0.033 |
| Self reward RDM | ACCg | 0.038 | 0.008 | <0.001 | <0.001 |
| | AI | 0.035 | 0.009 | <0.001 | 0.002 |
| | dACC/dmPFC | 0.041 | 0.009 | <0.001 | <0.001 |
| | TPJ | 0.026 | 0.009 | 0.005 | 0.008 |
| Other subjective value RDM | ACCg | 0.038 | 0.014 | 0.009 | 0.013 |
| | AI | 0.051 | 0.013 | <0.001 | <0.001 |
| | dACC/dmPFC | 0.073 | 0.011 | <0.001 | <0.001 |
| | TPJ | 0.044 | 0.012 | <0.001 | <0.001 |
| Self subjective value RDM | ACCg | 0.055 | 0.012 | <0.001 | <0.001 |
| | AI | 0.047 | 0.012 | <0.001 | <0.001 |
| | dACC/dmPFC | 0.064 | 0.012 | <0.001 | <0.001 |
| | TPJ | 0.026 | 0.011 | 0.018 | 0.024 |

Note. RDM: representational dissimilarity matrix, Mean: Kendall's $\tau_A$ correlations between brain RDMs and model RDMs, SE: standard error of the mean, FDR *p*: false-discovery rate corrected *p* value across 24 comparisons.

**Table S6. Whole-brain RSA searchlight results. Related to Figure 5 and Figure 6.**

| Brain Region | L/R | Peak voxel | | | k | t | z |
|---|---|---|---|---|---|---|---|
| **Conjunction: RSA Effort** | | | | | | | |
| Precuneus | R | 24 | -70 | 42 | 1359 | 5.55 | 5.06 |
| | R | 38 | -76 | 14 | | 4.96 | 4.60 |
| | R | 28 | -72 | 54 | | 4.26 | 4.02 |
| Precuneus | L | -18 | -74 | 50 | 409 | 4.55 | 4.26 |
| | L | -24 | -64 | 58 | | 3.92 | 3.73 |
| | L | -24 | -74 | 42 | | 3.85 | 3.66 |
| **Other > Self: RSA effort** | | | | | | | |
| *No suprathreshold voxels* | | | | | | | |
| **Self > Other: RSA effort** | | | | | | | |
| Postcentral gyrus | R | 18 | -46 | 66 | 340 | 4.84 | 4.49 |
| | R | 6 | -44 | 68 | | 4.06 | 3.85 |
| | R | 12 | -58 | 64 | | 3.72 | 3.55 |
| **Conjunction: RSA Subjective value** | | | | | | | |
| Precentral gyrus | L | -38 | -22 | 56 | 5941 | 4.85 | 4.50 |
| *ext. suppl. motor area* | L | -10 | -6 | 62 | | 4.83 | 4.49 |
| | L | -10 | 6 | 58 | | 4.80 | 4.46 |
| Superior frontal gyrus | L | -22 | 36 | 32 | 731 | 4.80 | 4.46 |
| | L | -22 | 46 | 30 | | 4.29 | 4.04 |
| *ext. dorsomedial prefrontal cortex* | L | -20 | 40 | 24 | | 4.27 | 4.03 |
| Anterior insula | L | -46 | 18 | 6 | 563 | 4.11 | 3.89 |
| *ext. Inferior frontal gyrus* | L | -56 | 16 | 22 | | 4.10 | 3.88 |
| | L | -32 | 30 | 10 | | 3.99 | 3.79 |
| Inferior parietal lobe | L | -48 | -46 | 50 | 278 | 4.02 | 3.81 |
| *ext. superior parietal lobe* | L | -32 | -56 | 56 | | 3.93 | 3.74 |
| | L | -40 | -58 | 56 | | 3.79 | 3.61 |
| **Other > Self: RSA subjective value** | | | | | | | |
| *No suprathreshold voxels* | | | | | | | |
| **Self> Other: RSA subjective value** | | | | | | | |
| Posterior cingulate | R | 20 | -20 | 50 | 578 | 4.78 | 4.45 |
| | R | 22 | -24 | 42 | | 4.76 | 4.43 |
| *ext. Posterior insula* | R | 32 | -24 | 24 | | 4.41 | 4.14 |
| Midbrain/ventral tegmental area | R | 4 | -22 | -16 | 291 | 4.44 | 4.16 |
| | L | -2 | -28 | -18 | | 4.28 | 4.04 |
| | L | -10 | -30 | -28 | | 3.75 | 3.57 |
| **Conjunction: RSA Reward** | | | | | | | |
| Precentral gyrus | R | 30 | -14 | 56 | 1058 | 5.40 | 4.94 |
| | R | 30 | -22 | 58 | | 4.90 | 4.55 |
| | R | 34 | -20 | 46 | | 4.58 | 4.29 |
| Cuneus | L | -12 | -76 | -22 | 2281 | 5.32 | 4.88 |
| *ext. lingual gyrus* | L | -20 | -82 | -14 | | 5.04 | 4.66 |
| | L | -10 | -86 | -14 | | 4.67 | 4.36 |
| Precentral gyrus | L | -42 | -14 | 60 | 1991 | 4.95 | 4.58 |
| *ext. inferior frontal gyrus* | L | -38 | 18 | 30 | | 4.42 | 4.15 |
| *ext. middle frontal gyrus* | L | -38 | 4 | 52 | | 4.39 | 4.12 |
| Superior parietal lobe | L | -22 | -66 | 58 | 319 | 4.45 | 4.18 |
| *ext. Inferior parietal lobe* | L | -28 | -52 | 48 | | 3.61 | 3.46 |
| Paracentral lobule | R | 2 | -42 | 58 | 285 | 4.30 | 4.05 |
| *ext. Precuneus* | L | -6 | -52 | 56 | | 3.59 | 3.44 |
| | L | -6 | -38 | 52 | | 3.52 | 3.38 |
| **Other > Self: RSA reward** | | | | | | | |
| *No suprathreshold voxels* | | | | | | | |
| **Self > Other: RSA reward** | | | | | | | |
| Precuneus | R | 34 | -78 | -40 | 312 | 4.59 | 4.29 |
| | R | 16 | -68 | -48 | | 4.54 | 4.25 |
| | R | 26 | -76 | -42 | | 4.32 | 4.07 |

Whole-brain searchlight results. For all regions, FWE P < 0.05 cluster-level whole-brain corrected after thresholding at p<.001. ext, extending into; k, cluster extent; L, left; PE, R, right.

**Table S7. Whole-brain univariate results. Related to Figure 5 and Figure 6.**

| Brain Region | L/R | Peak voxel | | | k | t | z |
|---|---|---|---|---|---|---|---|
| **Conjunction: univariate subjective value** | | | | | | | |
| Dorsal anterior cingulate cortex | R | 8 | 26 | 34 | 1359 | 5.16 | 4.75 |
| | L | -6 | 16 | 46 | | 5.14 | 4.74 |
| | L | -6 | 28 | 34 | | 4.70 | 4.38 |
| **Other > Self: univariate subjective value** | | | | | | | |
| *No suprathreshold voxels* | | | | | | | |
| **Self > Other: univariate subjective value** | | | | | | | |
| *No suprathreshold voxels* | | | | | | | |
| **Conjunction: univariate force required** | | | | | | | |
| Precentral gyrus | L | -20 | -28 | 64 | 116518 | 15.63 | >8 |
| *ext. cerebellum* | L | 2 | -70 | -38 | | 14.10 | >8 |
| | L | 2 | -60 | -12 | | 13.88 | >8 |
| **Other > Self: univariate force required** | | | | | | | |
| Posterior parietal lobe (temporo-parietal junction) | L | -50 | -62 | 40 | 776 | 5.28 | 4.85 |
| | L | -26 | -68 | 56 | | 3.57 | 3.42 |
| Superior frontal gyrus (dorsomedial prefrotnal cortex) | L | -12 | 26 | 52 | 2133 | 4.91 | 4.55 |
| | R | 12 | 48 | 38 | | 4.86 | 4.51 |
| | L | -16 | 50 | 28 | | 4.51 | 4.22 |
| Superior temporal gyrus | L | -56 | -38 | -8 | 414 | 4.51 | 4.22 |
| | L | -52 | -46 | -4 | | 4.32 | 4.07 |
| | L | -44 | -34 | -6 | | 4.18 | 3.95 |

Whole-brain univariate results for parametric modulators of subjective value (at offer) and force required (at onset of effort). For all regions, FWE $P < 0.05$ cluster-level whole-brain corrected after thresholding at $p<.001$. ext, extending into; k, cluster extent; L, left; PE, R, right.
