# Supplementary material for: Distinct neural representations for prosocial and self-benefiting effort
Source: Curr Biol. 2022 Oct 10;32(19):4172–4185.e7. doi: 10.1016/j.cub.2022.08.010 (PMC9616728; doi:10.1016/j.cub.2022.08.010)
Supplement: Document S2. Article plus supplemental information [file mmc2.pdf]

Article

Current Biology

Distinct neural representations for prosocial and self-benefiting effort

Highlights

- Prosocial behaviors frequently involve exerting effort
- Human participants completed an effort-based decision-making task during fMRI
- The anterior cingulate gyrus represented the effort costs of prosocial acts
- Ventral tegmental area and ventral insula represented value for oneself

Authors

Patricia L. Lockwood,
Marco K. Wittmann, Hamed Nili, ...,
Jo Cutler, Masud Husain,
Matthew A.J. Apps

Correspondence

p.l.lockwood@bham.ac.uk

In brief

Actions that help others—prosocial behaviors—are vitally important for reducing the challenges to humanity. However, helping others requires effort, and people are effort averse. Using fMRI, Lockwood et al. show a distinct representation of prosocial effort in the anterior cingulate gyrus when deciding, and when exerting force, to help.

Check for updates

Lockwood et al., 2022, Current Biology *32*, 4172–4185
October 10, 2022 © 2022 The Author(s). Published by Elsevier Inc.
https://doi.org/10.1016/j.cub.2022.08.010

CellPress

CellPress
OPEN ACCESS

Current Biology

Article

Distinct neural representations for prosocial and self-benefiting effort

Patricia L. Lockwood,[1,2,3,4,5,11,13,*] Marco K. Wittmann,[3,4,8,9] Hamed Nili,[4,6] Mona Matsumoto-Ryan,[3] Ayat Abdurahman,[3,4,7] Jo Cutler,[1,2,3] Masud Husain,[3,10] and Matthew A.J. Apps[1,2,3,5,12]
[1]Centre for Human Brain Health, School of Psychology, University of Birmingham, Birmingham B15 2TT, UK
[2]Institute for Mental Health, School of Psychology, University of Birmingham, Birmingham B15 2TT, UK
[3]Department of Experimental Psychology, University of Oxford, Anna Watts Building, Woodstock Road, Oxford OX2 6GG, UK
[4]Wellcome Centre for Integrative Neuroimaging, University of Oxford, John Radcliffe Hospital, FMRIB Building, Headington, Oxford OX3 9DU, UK
[5]Christ Church, University of Oxford, St Aldate's, Oxford OX1 1DP, UK
[6]Department of Excellence for Neural Information Processing, Center for Molecular Neurobiology (ZMNH), University Medical Center Hamburg-Eppendorf (UKE), Martinistraße 52, 20251 Hamburg, Germany
[7]Department of Psychology, University of Cambridge, Downing Place, Cambridge CB2 3EB, UK
[8]Department of Experimental Psychology, University College London, 26 Bedford Way, London WC1H 0AP, UK
[9]Max Planck UCL Centre for Computational Psychiatry and Ageing Research, University College London, Russell Square House 10-12 Russell Square, London WC1B 5EH, UK
[10]Nuffield Department of Clinical Neurosciences, University of Oxford, Oxford OX3 9DU, UK
[11]Twitter: @thepsychologist
[12]Twitter: @MSNlab
[13]Lead contact
*Correspondence: p.l.lockwood@bham.ac.uk
https://doi.org/10.1016/j.cub.2022.08.010

SUMMARY

Prosocial behaviors—actions that benefit others—are central to individual and societal well-being. Although the mechanisms underlying the financial and moral costs of prosocial behaviors are increasingly understood, this work has often ignored a key influence on behavior: effort. Many prosocial acts are effortful, and people are averse to the costs of exerting them. However, how the brain encodes effort costs when actions benefit others is unknown. During fMRI, participants completed a decision-making task where they chose in each trial whether to "work" and exert force (30%–70% of maximum grip strength) or "rest" (no effort) for rewards (2–10 credits). Crucially, on separate trials, they made these decisions either to benefit another person or themselves. We used a combination of multivariate representational similarity analysis and model-based univariate analysis to reveal how the costs of prosocial and self-benefiting efforts are processed. Strikingly, we identified a unique neural signature of effort in the anterior cingulate gyrus (ACCg) for prosocial acts, both when choosing to help others and when exerting force to benefit them. This pattern was absent for self-benefiting behaviors. Moreover, stronger, specific representations of prosocial effort in the ACCg were linked to higher levels of empathy and higher subsequent exerted force to benefit others. In contrast, the ventral tegmental area and ventral insula represented value preferentially when choosing for oneself and not for prosocial acts. These findings advance our understanding of the neural mechanisms of prosocial behavior, highlighting the critical role that effort has in the brain circuits that guide helping others.

INTRODUCTION

From holding open a door for a stranger to volunteering for a local charity, humans often make decisions to incur costs to benefit others.[1,2] Such "prosocial" behaviors are vital for maintaining individual physical[3] and mental health[4] and are positively correlated with economic success.[5] However, although a plethora of research has probed the psychological and neural mechanisms underlying how people make decisions about whether to donate to charity or share money, much of this work overlooks a key component: effort.[6–9] In order to behave prosocially, we have to decide whether we are willing to exert effort, and once committed, to energize our actions.[9,10] However, how the brain represents the effort of a prosocial act, and whether this is distinct from self-benefiting acts, is unknown. Understanding these distinctions is critical for connecting computational and neural explanations of social behavior.[11–13]

Effort is typically considered costly and aversive.[14–17] If two courses of action are associated with the same rewarding outcome, most individuals will choose the less effortful course. This phenomenon, referred to as effort discounting, relies on computations in which rewards are devalued by exerting effort.[9,18–20] As such, people only exert effort when it is "worth it" for reward. Research across species has begun to identify the anatomy

This is an open access article under the CC BY license (http://creativecommons.org/licenses/by/4.0/).

CellPress
OPEN ACCESS

engaged during such computations. Activity in the dorsal anterior cingulate cortex (dACC)/dorsomedial prefrontal cortex (dmPFC) and anterior insula (AI) has consistently been shown to covary with the magnitude of rewards and level of task difficulty, both prior to and during the performance of a task.[18,21–25] In addition, activity in these regions tracks subjective value during effort-based decisions.[18,24–31] Lesions to these areas have been linked to reductions in motivated behavior and a reduced willingness to exert effort.[32] These findings implicate the dACC/dmPFC and AI as crucial when deciding whether to exert effort for reward, and when energizing effortful processes. Although meta-analyses also highlight other areas such as ventral striatum and ventromedial prefrontal cortex (vmPFC) during effort-based decision-making,[28] these regions might predominantly encode reward and subjective value rather than effort per se.[18,20,33]

However, existing work has typically only examined self-benefiting behaviors, where the effort is exerted to obtain rewards for one's own benefit. However, the cost of effort may be different when it comes to prosocial acts. Lockwood and colleagues[9] required participants to make decisions about whether they would rather take a rest for small reward (1 credit) or exert physical effort (30%–70% of their max grip strength) to obtain higher rewards (2–10 credits). On half, the trials participants chose whether to exert the effort to obtain credits for themselves, but on the other half, the credits were delivered to an anonymous other person. Although people were willing to exert effort to obtain rewards for others, the effort cost was evaluated to be greater than when effort was self-benefiting, and participants were less willing to exert higher levels of effort for prosocial acts.[8,9,34,35] This differential weighting of effort costs into valuations raises the possibility that partially distinct neural mechanisms guide decisions of whether to exert effort for prosocial and self-benefiting behaviors.

Although there is limited research examining the neural mechanisms underlying prosocial effort, studies examining how we vicariously process others' rewards or efforts implicate a potentially "socially" specialized system.[36,37] Studies in which self and other trials are separated in the design allow questions about social specificity to be addressed.[12] In such experiments, a sub-region of the anterior cingulate cortex lying in the gyrus (ACCg) is implicated in processing social information. Neurophysiological recordings in monkeys indicate that the ACCg contains a higher proportion of neurons that signal exclusively when another, not oneself, receives rewards compared with other frontal regions.[38] ACCg response varies as a function of the vicarious net-value of other people exerting effort, the probability, and outcome of another person receiving a reward and tracks learning about others' ownership but does not process similar information about one's own effort, ownership, or reward.[39–42] Activity in ACCg has also been shown to correlate with self-reported individual differences in empathy, an affective process closely linked to motivating prosocial behaviors.[37,43,44] In addition, activity in a connected portion of the temporo-parietal junction (TPJ) has long been implicated in social cognition and prosocial behavior and encodes effort costs differently when behaviors switch from cooperation to competition.[45–51] Thus, a partially specialized neural circuit, comprising the ACCg and TPJ, may be engaged when deciding whether to exert effort to benefit others and applying the energy required.

Here, to address the question of whether prosocial efforts are processed distinctly from self-benefiting ones, participants completed a physical effort task[9] where self-benefiting and prosocial decisions were dissociated. People chose between a work option and rest option on each trial while undergoing functional magnetic resonance imaging (fMRI). Half of the trials were self-benefiting, where they chose whether to exert effort to obtain rewards for themselves (self), whereas the other half were prosocial—where the participant chose whether to exert effort to obtain rewards for an anonymous other person (other; STAR Methods; Figure 1). If they chose to work, they needed to execute the required force to obtain that reward. Using this design, we could examine activity time locked to the points in the trial where people made a decision to work or rest and responses during the exertion of force (Figure 1). Participants also completed a self-report assessment of empathy. We used a combination of parametric (and model-based) univariate analyses, as well as model-based, multivariate representational similarity analysis (RSA). RSA allowed us to test for social and self-specific representations of effort when deciding whether to benefit self and other, as well as subjective value and reward (Figure S1). This is crucial as RSA is based on the knowledge that population codes of neurons or voxels represent information a "neural population code."[52] These population codes cannot be captured in a univariate analysis that is based on the height of the BOLD signal, rather than the geometry (or similarity) between different experimental conditions.[12,53]

We show a distinct multivariate pattern of effort in the ACCg when deciding whether to act prosocially and that activity in this region scales parametrically with the force required during exertion in prosocial, but not self-benefiting acts. The strength of this pattern correlated with self-reported affective empathy and with the amount of force exerted into prosocial acts. A domain-general set of regions in the AI and dACC/dmPFC signaled multivariate and univariate representations of subjective value for self and other. In contrast, a ventral portion of the mid-insula and the ventral tegmental area (VTA) carried self-benefiting univariate and multivariate representations of subjective value, respectively. Together, these results reveal, behaviorally relevant, partially specialized neural mechanisms for prosocial and self-benefiting efforts.

RESULTS

People discount rewards by effort more strongly for others than for self

We analyzed how people's decisions to select the work offer over "rest" were affected by the effort required, reward on offer, and whether participants treated prosocial decisions as distinct from self-benefiting ones (recipient). We observed significant recipient*effort and recipient*reward interactions showing that people were less willing to help others at higher effort levels (OR = 1.20, 95% CI = [1.03, 1.40], $p = 0.01$) and lower reward levels (OR = 1.31 [1.11, 1.55], $p = 0.003$). We also observed main effects of recipient, effort, and reward (Figures 2A, 2B, and S2; Table S1). Therefore, participants were less willing to exert effort to reward other people than themselves. Participants also took longer to choose between work and rest when rewards were for another person (other mean = 1.16 s versus self mean = 1.07 s , $Z = -4.62$, $r = 0.19$ [0.02, 0.41], $p < 0.001$; Table 1).

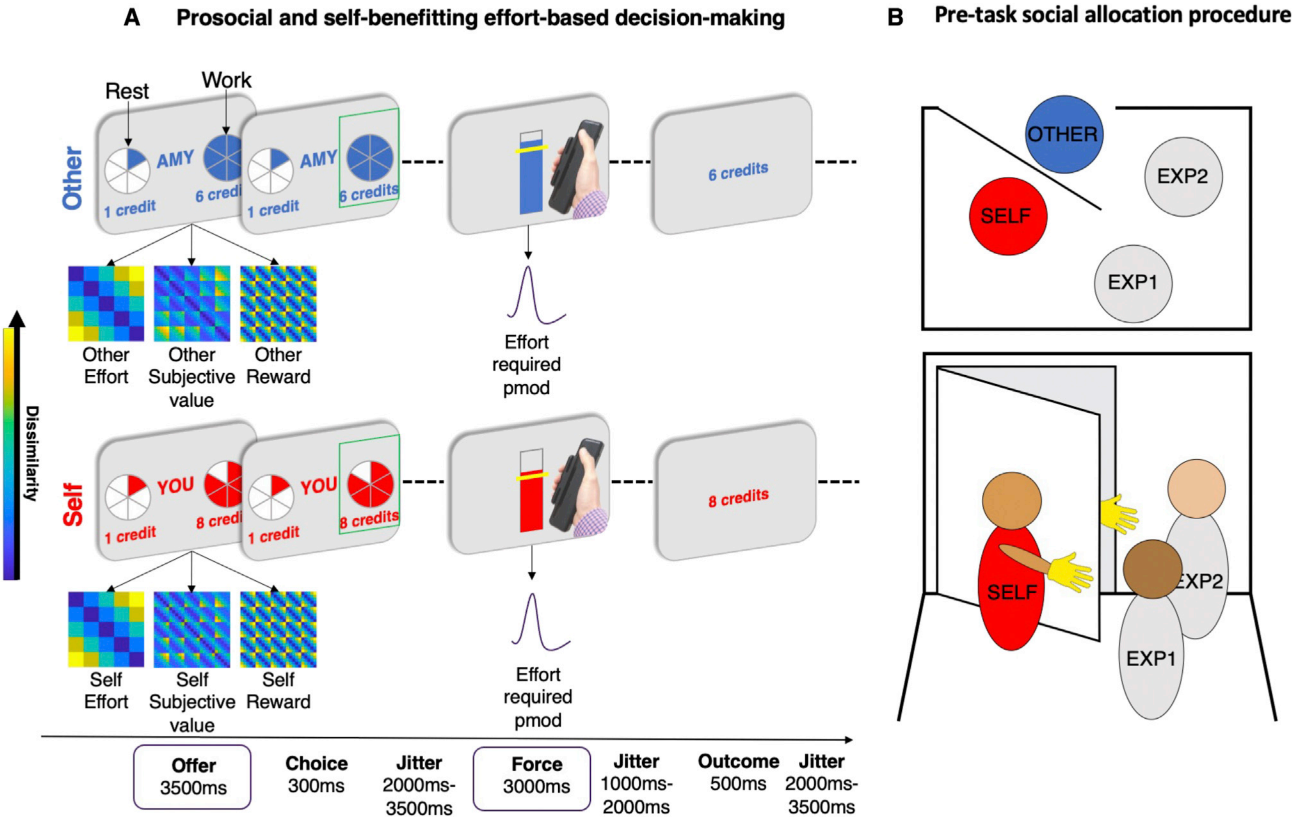

Figure 1. Prosocial and self-benefiting effort decision-making task

(A) Before undergoing fMRI, participants were instructed to squeeze as hard as they could to measure their maximum voluntary contraction (MVC) on a handheld dynamometer to threshold each effort level to their own strength. After thresholding and practice, participants were presented on each trial with a choice between a rest option, which required no effort (0% MVC, corresponding to one segment of the pie chart) for a low reward of 1 credit, and a work option, which required more effort (30%–70% MVC, corresponding to 2–6 segments in the pie chart) yet also generated more reward (2–10 credits). The offered reward and effort levels were orthogonal in the design. After making their selection, participants then had to exert the required force to the correct degree to receive the reward. Visual feedback of the amount of force used was displayed on the screen. Participants were informed that they would have to reach the required force level (marked by the yellow line) for at least 1 s of a 3 s window. Participants then saw the outcome that corresponded to the offer they had chosen, unless they were unsuccessful, in which case "0 credits" was displayed. Crucially, on self-trials, participants made the choice, exerted the effort, and received the reward themselves, whereas on other trials ("AMY" in this example), participants made the choice and exerted the effort, but the other participant received the reward. Self and other trials were interleaved (STAR Methods). Six 25 × 25 (5 effort and 5 reward levels) model representational dissimilarity matrices (RDMs) were constructed at the offer stage (STAR Methods; Figure S1). To examine activity during the force period, univariate parametric modulators (pmod) of the effort required on each trial were fitted to the onset of the force period. GLMs were inspected to ensure conditions and time points could be estimated independently with minimal correlations (STAR Methods; Figure S6). Yellow colors show conditions are more dissimilar, whereas dark blue colors show conditions are more similar in terms of the Euclidean distance between conditions.

(B) Participants were designated as "Player 1" (self) and told that they would be making decisions that impacted another player "Player 2" (other) who they met at the beginning of the testing session with their identity obscured (to control for influences of identity or reciprocity; STAR Methods). The procedure involved 4 people, two experimenters, EXP1 and EXP2, and two participants, self and other.

See also Figures S1 and S2.

Next, we fit and compared a range of different models of effort discounting to participants' choice behavior using maximum likelihood estimation (STAR Methods).[9,18–20,35] These models tested different theoretical predictions regarding the effect of effort on rewards in the task (whether discounting was linear, hyperbolic, or parabolic). We also considered additional classes of models with either free parameters on reward (in addition to effort), reward only, or reward and effort difference, but these models showed poor identifiability and worse fit (Figure S3); hence, they were not evaluated further. The winning model in the majority of participants (66%) was a parabolic model with separate discount parameters (K_{self} and K_{other}) and a single noise parameter (β), (STAR Methods; Figures 2C–2F). The $2K1\beta$ model also won in the majority of participants compared with other closely performing models in terms of Bayesian information criterion (BIC) scores (Figures 2C and S3C). We further validated our winning model in four ways. First, we calculated the median R^2 and found that the model was able to explain 92% (SD = 10%) of the variance of choices. Second, we performed model identifiability analyses[54] using simulated data and showed that our model comparison procedure accurately selected the correct winning model with high identifiability (STAR Methods; Figures S3A and S3B). Third, we calculated the balanced accuracy for our winning

CellPress
OPEN ACCESS

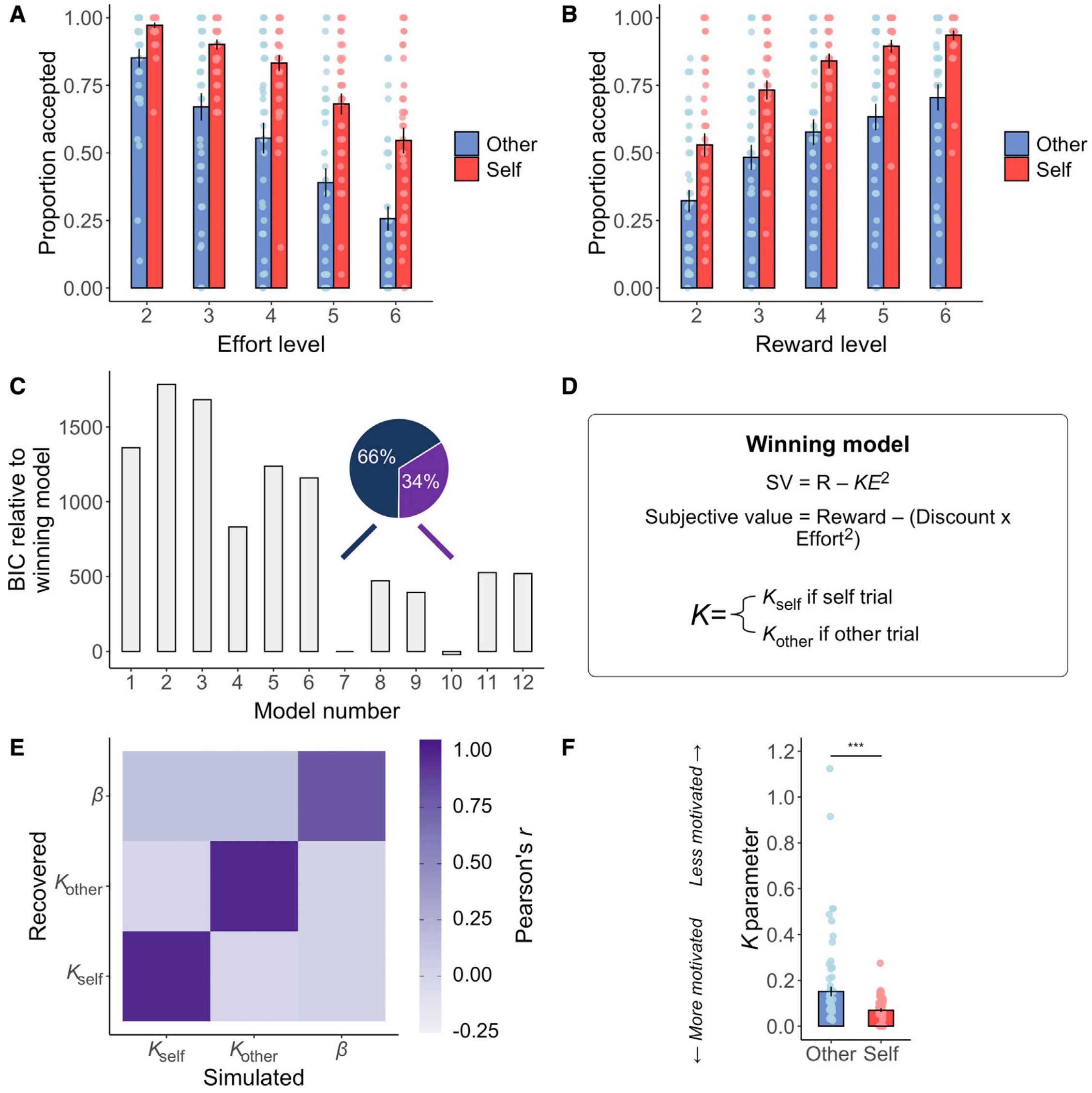

Figure 2. Choice and computational modeling of prosocial and self-benefiting decisions

(A) Participants were less willing to accept the work offer over the rest offer as the effort level increased, particularly when working to benefit someone else ($p = 0.01$).

(B) The proportion of work offers accepted over the baseline option increased as reward increased, but this was less so when rewards were for other, compared with self ($p = 0.003$). Data are represented as mean ± SE.

(C) We compared a range of established computational models of effort discounting that varied in terms of whether models had a single or separate discount (K) parameter(s) for self and other trials (models 1–6 versus models 7–12) and whether the shape of the discount function was parabolic (models 1, 4, 7, and 10), linear (models 2, 5, 8, and 11), or hyperbolic (models 3, 6, 9, and 12). Model 7, which contained a single choice stochasticity parameter (β), explained behavior in the majority of participants and was selected as the winning model (STAR Methods). Bars show model BIC, proportions show the number of participants with the lowest BIC for model 7 compared with model 10. We also considered additional classes of models with either free parameter on reward (in addition to effort), reward only, or reward and effort difference, but these models showed poor identifiability and worse fit (Figure S3).

(D) Equation for the winning parabolic model with separate discount (K) parameters and a single choice stochasticity (β) parameter that explained behavior in the majority of participants.

(E) Parameter recovery using simulated data from the winning model and choice schedule showed excellent recovery.

(F) Statistical comparison of the K parameters from model 7 showed that participants had a lower K parameter for self-benefiting compared with prosocial choices. Data are represented as median ± SE, ***$p < 0.001$, Wilcoxon two-sided signed rank test.

See also Figure S2 and S3 and Table S1.

model, which was high (83%). Finally, parameter recovery[54,55] showed recoverable parameters based on our schedule (K_{self} = 98%, K_{other} = 98%, β = 80%; Figure 2E).

Comparing discount parameters for self and other from the winning model showed significantly higher K values for other (median = 0.15) than for self (median = 0.07, $Z = -5.34$,

Table 1. Behavioral variables compared between self and other trials

	Self mean	Self SE	Other mean	Other SE	Z	*r*	CI low	CI up	p
Accept	0.79	0.03	0.54	0.04	−4.73	0.47	0.25	0.62	<0.001
RT	1.07	0.03	1.16	0.04	−4.62	0.19	0.01	0.4	<0.001
TiW	1.86	0.03	1.86	0.03	−0.62	0.01	0	0.27	0.54
Success	0.97	0.01	0.97	0.01	−0.88	0.05	0	0.29	0.38
Force	0.64	0.01	0.58	0.01	−4.77	0.35	0.14	0.54	<0.001

RT, reaction time; TiW, time in window over the required force level; *r*, standardized effect size; CI, 95% confidence interval for odds ratio; low, lower CI; up, upper CI; values are from Wilcoxon two-sided rank tests comparing self and other.

$r = 0.50$ [0.30, 0.65], $p < 0.001$; Figure 2F). Thus, as the required effort increased, the subjective value of decisions decreased at a higher rate when making prosocial versus self-benefiting choices.

People exert less force when deciding to help others

A second critical aspect of helping others is that after we have decided to help, we have to energize our actions, and we have to exert the effort required. In addition to being less motivated in choosing to put in effort for others, people may be less invigorated and exert less force particularly at higher effort levels.[9,35] We used a linear mixed-effects model (LMM) to predict the force that participants exerted on each trial as a function of effort, reward, and their interactions (STAR Methods). The amount of required force a participant exerted on each trial was precisely signaled on the screen, and real-time feedback showed whether they were achieving the required force level. Thus, for these analyses, the raw (rather than squared) effort levels were used as a predictor. We observed a significant three-way interaction between effort, reward, and recipient ($\chi^2_{(16)} = 42.03$, $p = 0.002$). We also found significant interactions between recipient and reward ($\chi^2_{(4)} = 13.21$, $p = 0.01$), effort and reward ($\chi^2_{(16)} = 49.88$, $p = 0.001$), and main effects of recipient, effort, and reward (all $\chi^2 > 7.09$, all $p < 0.001$; Figures 3A and 3B; Table S2). Importantly, there was no significant difference in success (exerting effort for at least 1 second of a 3-s period, fixed for each trial) between self (mean = 0.97) and other trials (mean = 0.97, $Z = -0.88$, $r = 0.05$ [0.00, 0.29], $p = 0.38$; Table 1) and Bayesian evidence for no difference ($BF_{01} = 4.19$, substantial evidence in support of the null). Self and other trials also did not differ in the length of time participants maintained the required level of effort (self mean = 1.86 s, other mean = 1.86 s, $Z = -0.62$, $r = 0.01$ [0.00, 0.25], $p = 0.54$; Table 1; $BF_{01} = 5.61$, substantial evidence in support of the null). Finally, we correlated the difference in reaction times (RTs) for choosing to work versus rest for self and other and the difference in the amount of time that effort was maintained over the line. The association was positive but not significant ($r_{(36)} = 0.28$, $p = 0.09$). Therefore, participants applied less force for other-benefiting than self-benefiting decisions, particularly at high effort levels, but were not less successful.[9,35]

Prosocial and self-benefiting neural computations

Having established robust behavioral differences consistent with prior work,[9,34,35] we next examined whether there were distinct or common neural processes involved using a multivariate, RSA approach.[53,56] RSA can complement and add to inferences that are made based upon univariate fMRI analyses or multivariate approaches that distinguish dichotomous variables. RSA analyses have similarities to population analyses applied to neurophysiological recordings. As such, they can be used to link together the algorithmic and implementational levels of explantation.[12,57] RSA is well suited to designs where stimuli can be processed along continuums in different dimensions.[58] Therefore, it was ideal for this experiment where the work offers can be parametrized in terms of effort level, reward level, or subjective value. RSA can be more sensitive than univariate analysis since it captures effects that are washed away by averaging across voxels[59] but that are crucial for understanding social specialization.[53,60]

We calculated brain representational dissimilarity matrices (RDMs) coding for dissimilarity (correlation distance[59]) in multivariate patterns of voxels for all pairs of conditions. This resulted in a 25 × 25 matrix computed separately for self and other trials (Figure 1; STAR Methods). We created six model RDMs, which reflected the dissimilarity in self-effort, other effort, self-subjective value and other subjective value (from the winning computational model), and self reward and other reward. Inferences were drawn by correlating each model RDM with each brain RDM using Kendall's τ_A.[56] Brain RDMs were calculated using both a hypothesis driven, anatomically specific region-of-interest (ROI) approach (see below) and a whole-brain data-driven searchlight approach (STAR Methods).

In addition to the multivariate approach, we conducted two univariate analyses. The first used the trial-by-trial subjective values from the best-fitting computational model at the time of choice. The second examined activity time locked to the force period, which scaled with the amount of effort required (Figure 1).

The aim of this study was to test specific hypotheses about regions that have previously been linked to guiding effort-based decisions and those linked to processing social information that could guide prosocial behaviors. Given extensive previous work on the neural systems involved in social decision-making[12,36,61] and self-relevant effort-based decision-making,[18,24–31] we focused our fMRI analysis on four ROIs where we had strong a priori hypotheses using independent anatomical masks defined using pre-existing parcellations (see below). These allowed us to probe distinct contributions of different portions of the cingulate cortex, distinguishing dorsal dACC/dmPFC (area 8 m)[18,24,28] from more ventral portions of the ACCg[12,36,37,61] (Figure S4; STAR Methods). We also used these masks for labeling of activations in whole-brain and univariate analyses. In addition, we conducted exploratory ROI analyses in vmPFC (areas 11 and 14 m[62]) and ventral striatum (Harvard-Oxford Atlas) (STAR Methods; Figure S5; Tables S3 and S4).

CellPress
OPEN ACCESS

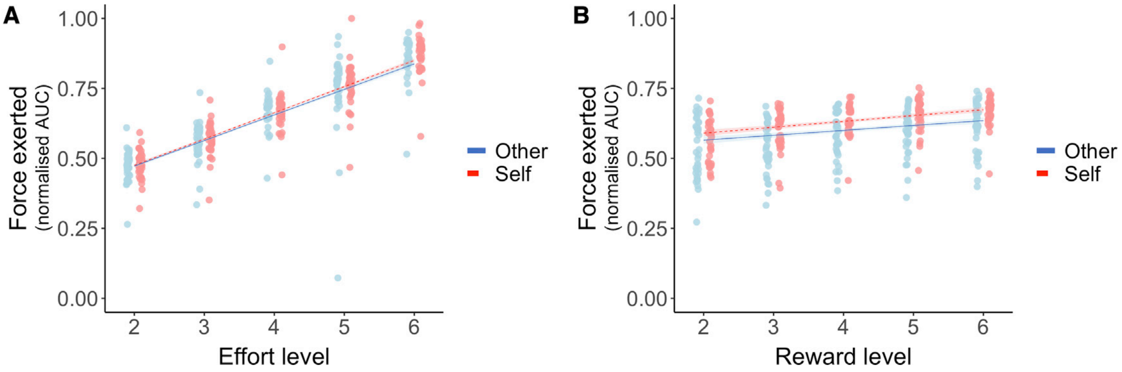

Figure 3. Force exerted as a function of effort level and reward level for self and other

(A) Force exerted (normalized areas under the curve during the effort period) for each level of effort. Participants exerted less force for others overall, and there was a significant three-way interaction between recipient, effort, and reward.

(B) Force exerted for each reward level shows that participants exerted more force for higher rewards, but this effect was reduced when the other person would benefit. Error bars show standard error.

See also Table S2.

Patterns of prosocial effort in ACCg

For a region to be considered coding prosocial, and not self-benefiting, effort, its RDM should correlate with the other-effort model RDM, and not with the self-effort model RDM, and there should be a significant difference between the strength of those correlations. This would demonstrate that the neural patterns discriminate strongly between task conditions that vary in the levels of effort that are required to be put in for another person; but the same patterns do not vary with the differences in effort level when the decisions are about oneself. In line with our hypothesis, the ACCg ROI carried a multivariate representation of effort on prosocial trials—other-effort mean rank correlation $\tau_A \pm$ SE: ACCg = 0.026 ± 0.009, $p = 0.005$, surviving FDR correction for 24 comparisons (6 models, 4 brain areas, 2 recipients)—and was the only ROI to display a significant difference between the other-effort and self-effort RDMs ($Z = -2.73$, effect size r = 0.44 [0.13, 0.69], $p = 0.006$; Figure 4A).

Multivariate patterns in our three other ROIs also showed significant correlations with the other-effort RDM when making prosocial choices (other-effort mean rank correlation $\tau_A \pm$ SE: TPJ = 0.033 ± 0.010, $p = 0.001$; AI = 0.021 ± 0.008, $p = 0.006$; dACC/dmPFC = 0.029 ± 0.008, $p = 0.001$). In contrast for self-effort patterns, only the TPJ brain RDM significantly correlated with the self-effort model RDM (self-effort mean rank correlation $\tau_A \pm$ SE: ACCg = 0.002 ± 0.009, $p = 0.61$; TPJ = 0.024 ± 0.010, $p = 0.026$; AI = 0.008 ± 0.009, $p = 0.40$; dACC/dmPFC = 0.016 ± 0.010, $p = 0.16$). Critically, although TPJ, AI, and dACC/dmPFC also represented prosocial effort, they did not do so more strongly than for the self-effort RDMs (Wilcoxon two-sided signed-rank test, all $p > 0.07$). Thus, although all ROIs showed significant correlations between the brain and model RDMs for the other-effort condition, it was only in the ACCg that showed a stronger pattern, relative to the self-benefiting condition.

Notably, the specificity for prosocial effort in the ACCg was not due to total differences for other and self-representations, as the ACCg represented other and self-offers as equally dissimilar (STAR Methods; Bayesian paired sample t test $BF_{01} = 4.61$, substantial evidence in support of the null). A representational connectivity analysis[64] suggested that ACCg representations on other trials correlated with dACC/dmPFC and AI and more strongly than on self trials (see STAR Methods for full details). Further evidence for the specificity of ACCg for prosocial effort also came from examining representations of other reward. Although patterns in several regions significantly correlated with the other-reward RDM (other-reward mean rank correlation $\tau_A \pm$ SE: ACCg = 0.009 ± 0.007, $p = 0.16$; TPJ = 0.016 ± 0.007, $p = 0.027$; AI = 0.020 ± 0.008, $p = 0.006$; dACC/dmPFC = 0.025 ± 0.007, $p = 0.001$), no region significantly represented others' rewards more strongly than self rewards (Table S5). Thus, multivariate patterns in the ACCg represented effort costs specifically when making prosocial but not self-benefiting choices (see Table S6 for exploratory whole-brain searchlight results).

Parametric modulation of effort level when exerting force for others in ACCg

We next used univariate analysis to determine regions in which activity scaled with the required effort level during the force period. We found that the BOLD response in an ACCg cluster fully overlapping with our anatomical ROI positively covaried with force for others ($x = 4$, $y = 2$, $z = 36$, $Z > 8.00$, $k = 455$, $p = 0.001$, family-wise error [FWE]-corrected), and within this cluster, a partially overlapping sub-cluster also showed a significant effect coding force for other greater than self ($x = -6$, $y = 24$, $z = 20$, $Z = 3.28$, $k = 41$, $p = 0.029$, FWE-small volume corrected [SVC]; Figure 4B). Analysis of the force period showed that at the whole-brain level, the left TPJ positively tracked force exerted for others more than self ($x = -50$, $y = -62$, $z = 40$, $Z = 4.85$, $k = 790$, $p < 0.001$, FWE-whole brain), with activation for other greater than self on the right side at small-volume corrected levels ($x = 52$, $y = -56$, $z = 40$, $Z = 3.65$, $k = 29$, $p = 0.046$, FWE-SVC). A region in the bilateral middle insula ($x = -38$, $y = 0$, $z = 12$, $Z = 3.96$, $k = 105$, $p = 0.009$, FWE-SVC; $x = 44$, $y = 4$, $z = 10$, $Z = 3.65$, $k = 83$, $p = 0.027$, FWE-SVC) tracked both self and other force but responded more strongly to other. Outside of our ROIs, we also observed significant tracking of force for other more than self in a region of the

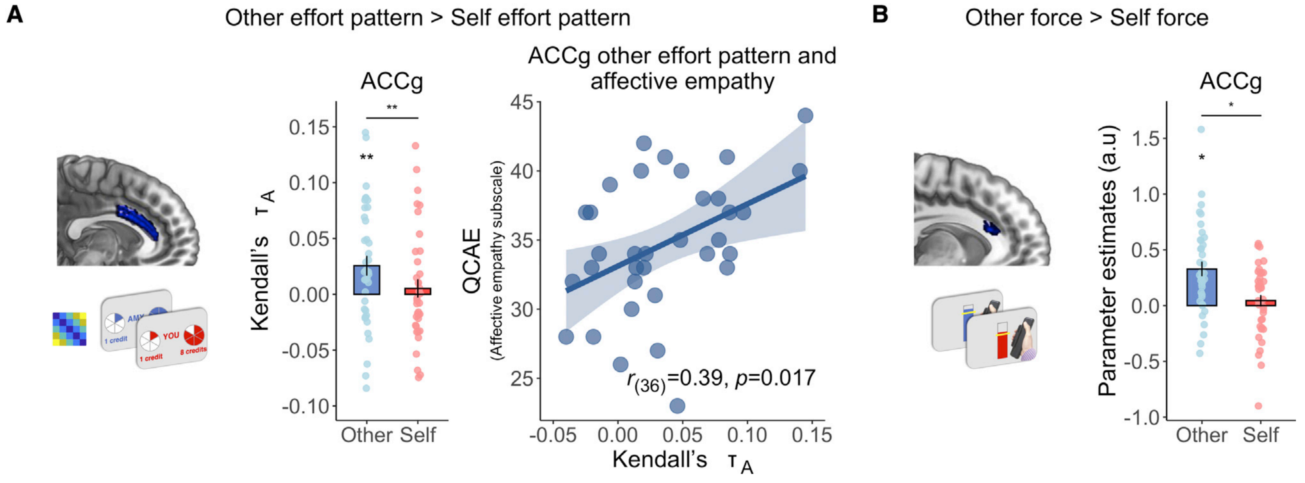

Figure 4. ACCg codes patterns of effort for others only, varies with level of affective empathy, and tracks effort required to benefit others only
(A) Across an independent structural ROI of the anterior cingulate gyrus (Neubert et al.[62]), multivoxel patterns of effort were encoded specifically for others. Kendall's τ_A indicates the extent to which the effort model RDM explains pattern dissimilarity between voxels in ACCg. ACCg shows a significant correlation between the effort RDM and brain RDM for other, and a greater correlation between the brain RDM and effort RDM for other compared with self. Variability in ACCg effort patterns for other was explained by individual difference in affective empathy, as measured by the Questionnaire for Cognitive and Affective Empathy (QCAE[63]). In contrast, there was no significant correlation with cognitive empathy, and the two correlations were significantly different from one another.
(B) A univariate analysis time locked to the onset of the force period showed a cluster within the ACCg ROI that tracked amount of effort required specifically when making prosocial decisions ($x = -6$, $y = 24$, $z = 20$, $Z = 3.28$, $k = 41$, $p = 0.029$, FWE-SVC). Activation overlaid on an anatomical scan of the medial surface. *$p < 0.05$, **$p < 0.01$, and ***$p < 0.001$. Error bars show standard error.
See also Figures S2 and S4 and Table S5.

superior frontal gyrus extending into the paracingulate cortex and middle temporal gyrus (Table S7). No brain areas significantly responded more to the contrast self force greater than other force at the whole-brain level or in any of our ROIs.

Therefore, although several regions processed information about prosocial efforts, only the ACCg was more specialized. Multivariate patterns in the ACCg specifically encoded representations of prosocial effort when making a choice, and univariate signals scaled with how much effort was required, with no such signals for self-benefiting efforts.

Multivariate representations of prosocial effort in ACCg correlate with individual differences in affective empathy and force exerted for others

Multiple lines of evidence suggest that empathy is associated with prosocial behavior and social cognition more broadly,[37,41,43,65,66] specifically because these constructs are hypothesized to be closely related.[37,66,67] Previous work shows that empathy and associated constructs (lack of empathy in psychopathy) are correlated with the willingness to exert effort to benefit others in large samples[9,34] and variability in ACCg response to social information correlates with empathy.[37,41,68] An important distinction is often made in the literature between "affective empathy," resonating with the affect of others, and "cognitive empathy," understanding the thoughts and affective states of others.[63] Thus, we next sought to evaluate whether multivariate and univariate signals of prosocial effort varied with individual differences in empathy. Since we found evidence for specific effort patterns during prosocial acts in ACCg only, we focused our analysis on responses in this region. We found that affective empathy was positively correlated with the strength of prosocial effort patterns in ACCg (Pearson's $r_{(36)} = 0.39$, $p = 0.02$), whereas cognitive empathy was not (Pearson's $r_{(36)} = 0.05$, $p = 0.78$, correlations significantly different $t = 2.04$, $p = 0.02$). Participants who were higher in affective empathy also exerted more force to gain rewards for others (Pearson's $r_{(36)} = 0.34$, $p = 0.04$), which was not the case for cognitive empathy (Pearson's $r_{(36)} = 0.17$, $p = 0.31$), although correlations were not significantly different $t = 0.95$, $p = 0.17$).

The level of force exerted on other trials in turn was positively associated with the strength of prosocial effort patterns in ACCg (Pearson's $r_{(36)} = 0.38$, $p = 0.02$). There were no significant associations between affective empathy and proportion to work for other (Pearson's $r_{(36)} = 0.09$, $p = 0.59$) or accepting work versus rest for other compared with self (Pearson's $r_{(36)} = 0.15$, $p = 0.38$). For the univariate tracking of prosocial effort during force, neither affective or cognitive empathy were significantly correlated (all $r > -0.18$, all $p > 0.29$). Therefore, individuals who reported being more affectively empathic represented the effort of behaviors more distinctly in the ACCg when deciding whether to act prosocially.

Next, we conducted exploratory analyses examining whether the proportion of decisions to help others, and amount of force subsequently exerted, related to univariate neural responses to force for others and multivariate patterns of prosocial effort, respectively. When linking these behaviors to neural responses, we focused on time points in the trial that were as independent as possible from the behavior, given the statistical issues with correlating individual behavior and neural signals.[69]

We found that ACCg representations of others effort positively correlated with amount of force exerted for other (Pearson's $r_{(36)} = 0.38$, $p = 0.018$; Figure S2D). ACCg univariate responses

CellPress
OPEN ACCESS

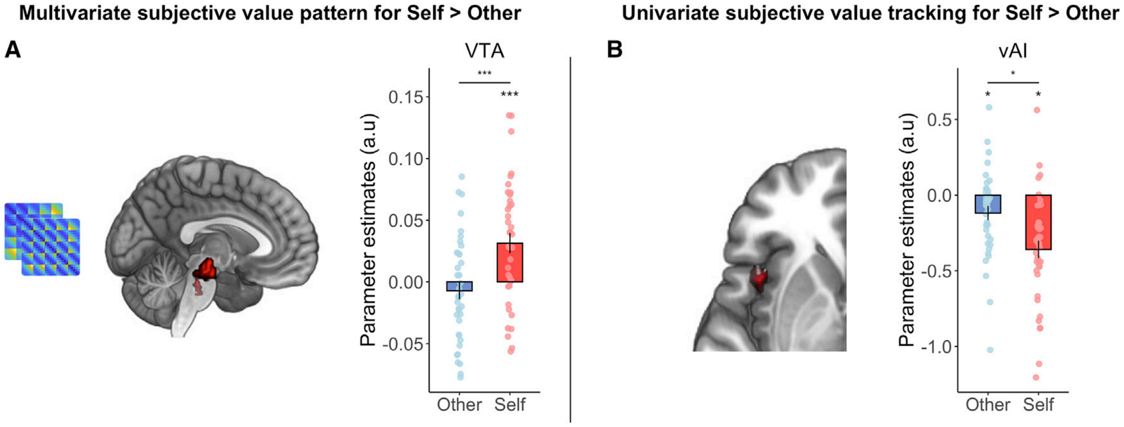

Figure 5. Self-benefiting and domain-general representations and tracking of subjective value

(A) A cluster putatively in the ventral tegmental area (VTA) encoded representational patterns of subjective value exclusively on self-benefiting trials (x = 4, y = −22, z = 16, k = 291, Z = 4.45, p = 0.03, FWE-whole brain corrected after thresholding at p < 0.001).

(B) A sub-region of the ventral anterior insula (vAI; x = −44, y = 10, z = −10, Z = 3.72, k = 59, p = 0.04, FWE-small volume) tracked subjective value of the chosen offer trial-by-trial more strongly for self-benefiting than other-benefiting choices.

Error bars show standard error.

See also Figure S4 and Tables S6 and S7.

to force exerted for other negatively correlated with proportion of choices to benefit other (Pearson's $r_{(36)} = -0.38$, p = 0.018; Figure S2E). Together, these results suggest that individuals with stronger patterns of others effort in ACCg were higher in empathy and exerted more subsequent force into prosocial acts.

Specific coding for self-benefiting acts in the midbrain and AI

Do any regions specifically code self-benefiting acts when making effort-based decisions? None of our ROIs showed a stronger correlation of the self-effort than other-effort RDM, and similarly for the SV RDM, no region showed a significantly stronger correlation for self than other (all Z < 1.37 || > 1.56, all p > 0.12; see Table S5 for reward RDM results). However, a whole-brain exploratory searchlight analysis revealed a significantly stronger correlation with the self-SV than other-SV model RDM in the midbrain, putatively in the VTA (x = 4, y = −22, z = 16, k = 291, Z = 4.45, p = 0.033, FWE-whole brain; Figure 5A; Table S6) and the posterior cingulate (x = 20, y = −20, z = 50, Z = 4.78, k = 578, p = 0.002, FWE-whole brain corrected; Table S6). The univariate analysis revealed a cluster in a ventral portion of the left AI (vAI; x = −44, y = 10, z = −10, Z = 3.72, k = 59, p = 0.04, FWE-SVC) in which activity scaled more strongly with SV when making self-benefiting than other-benefiting choices (Figure 5B). This cluster did not overlap with one that signaled SV on both self and other trials (Figure S4C). Such findings suggest that the VTA is engaged exclusively in making choices about exerting effort to benefit oneself, and vAI tracks subjective value more closely during self-benefiting than other-benefiting decisions.

Domain-general multivariate and univariate signals of subjective value for self and other

Previous research using univariate approaches has repeatedly implicated the dACC/dmPFC and AI in signaling SV during self-benefiting, effort-based choices in a domain-general manner.[18,24,28] Based on this work, we tested whether these regions contain information about SV when making both self-benefiting and prosocial choices (STAR Methods). We found significant correlations between the self-SV and other-SV RDMs in dACC/dmPFC and AI (self-SV mean rank correlation τ_A ± SE: dACC/dmPFC = 0.063 ± 0.012, p < 0.001; AI = 0.047 ± 0.012, p < 0.001; other-SV mean rank correlation τ_A ± SE: dACC/dmPFC = 0.073 ± 0.012, p < 0.001; AI = 0.051 ± 0.013, p < 0.001; all survive FDR correction; Figure 6A). Moreover, univariate conjunction analysis also revealed activity covarying with SV on self and other trials in dACC/dmPFC (x = 8, y = 26, z = 34, Z = 4.75, k = 1,033, p = 0.016, FWE-whole brain; Figure 6B) and bilateral AI (left: x = −28, y = 22, z = 6, Z = 4.47, k = 306, p < 0.001, FWE-SVC; right: x = 34, y = 24, z = 2, Z = 4.38, k = 222, p = 0.002, FWE-SVC; Figure 6B) that overlapped with the same portions of dACC/dmPFC and AI that coded the multivariate pattern. This is striking, given that the correlation distance is invariant to the mean activation level across voxels,[53,70] rendering the multivariate and univariate predictions of neural response separate (STAR Methods).

Outside of dACC/dmPFC and AI, we found multivariate patterns of subjective value that overlapped between self and other in TPJ and ACCg (self-SV mean rank correlation τ_A ± SE: TPJ = 0.026 ± 0.011, p = 0.018; ACCg = 0.055 ± 0.012, p < 0.001; other-SV mean rank correlation τ_A ± SE: TPJ = 0.044 ± 0.011, p < 0.001; ACCg = 0.038 ± 0.014, p = 0.009; all survive FDR correction). At the whole-brain level, searchlight analysis also showed responses in the superior frontal gyrus, inferior parietal lobe, and precentral gyrus in conjunction analyses (Table S6). No other areas significantly tracked self and other SV in any of our ROIs or at the whole-brain level. For effort, domain-general patterns were observed in the bilateral precuneus (Table S6), and for reward, in bilateral precentral gyrus, cuneus, and paracentral lobule (Table S6). Univariate conjunction analysis of effort required during the force period demonstrated wide-ranging activation at the whole-brain level, centering on the precentral gyrus and cerebellum (Table S7).

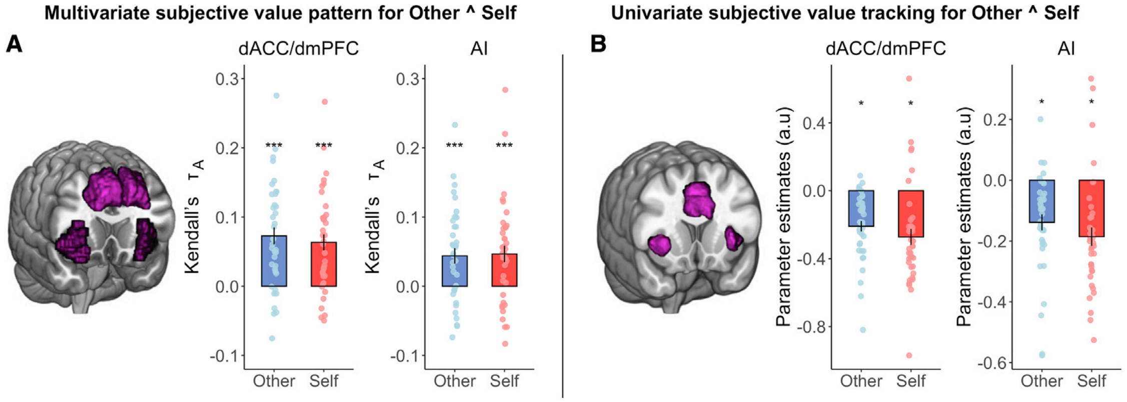

Figure 6. Multivariate and univariate patterns and signals of subjective value overlap in dACC/dmPFC and AI

(A) The dACC/dmPFC and AI showed significant correlations between the brain RDM and subjective value RDM pattern for both other and self-offers, consistent with a domain-general response in these regions.

(B) Univariate analysis also showed trial-by-trial tracking of subjective value in dACC/dmPFC (x = 8, y = 26, z = 34, Z = 4.75, k = 1,033, p = 0.016, FWE-whole brain) and AI (left: x = −28, y = 22, z = 6, Z = 4.47, k = 306, p < 0.001, FWE-SVC; right: x = 34, y = 24, z = 2, Z = 4.38, k = 222, p = 0.002, FWE-small volume) for both self and other. *p < 0.05, **p < 0.01, and ***p < 0.001. Error bars show standard error.

See also Figure S4 and Tables S6 and S7.

DISCUSSION

Many prosocial acts are effortful. However, the neural mechanisms that underlie how people decide whether to exert effort into prosocial acts and whether such mechanisms are distinct from self-benefiting acts are poorly understood.[6] Here, we show that the ACCg processes information that is crucial for making effort-based decisions when they are prosocial, but not when they are self-benefiting. The ACCg carried a multivariate representation of effort when deciding whether to help others and showed a univariate response to the degree of effort required while energizing prosocial acts. The representation of effort in this area was also stronger in individuals higher in self-reported empathy and in those who subsequently exerted more force into prosocial acts. In addition, we found a region in the midbrain which processed information only when making self-benefiting effort-based choices, a portion of the ventral AI that tracked self-subjective value more closely than other subjective value, as well as domain-general representations of SV in the dACC/dmPFC and distinct portions of bilateral AI. These findings highlight the importance of effort for understanding the neural mechanisms of prosocial and self-benefiting behaviors and that multivariate patterns and univariate model-based signals differentiate between prosocial and self-benefiting effortful acts.

There is a growing body of evidence to suggest that the ACCg is a vital cingulate sub-region for social cognition and vicariously processing information about others.[36,37] In macaques, lesions to ACCg, but not neighboring sub-regions, reduce sensitivity to social stimuli.[71] Neurophysiological recordings indicate that ACCg contains a higher proportion of neurons that respond exclusively when seeing others obtain a reward, but not when getting rewarded oneself.[38] In rodents, a putatively homologous region contains neurons that respond when seeing another receiving electrical shocks and when exerting competitive efforts.[72,73,74] In humans, single-unit recordings have identified that ACCg contains neurons that signal outcomes being delivered when learning from others', but not one's own reward, prediction errors.[75] In addition, neuroimaging studies have shown that this region responds to cues that are predictive of rewarding outcomes for others and not self, signals the value of others' rewarding outcomes but not one's own when they will have to exert effort for them, and encodes social prediction errors at the time of the outcomes of others actions.[36,37,42] Combined, this work suggests that the ACCg processes information about others that it does not process about ourselves.

Crucially, we found other-specific effort effects in the ACCg that went beyond vicarious processing of others' outcomes and extended them to prosocial behaviors. Such findings suggest that vicarious signals in the ACCg may not be epiphenomenal or simply reflecting one's emotional responses to others outcomes, but instead drive behavior.[11,12] In particular, the ACCg may be crucial for motivating people to help others and overcome barriers to others receiving positive outcomes. Consistent with this, we found representations of effort in the ACCg both when making prosocial decisions and when energizing prosocial but not self-benefiting actions.

Our finding fits with the notion that ACCg patterns do not simply reflect the reduced willingness to put in effort for others, since similar self-benefiting effort representations were absent in ACCg and those higher in empathy represented the distinction between effort costs more strongly. However, an alternative explanation is that ACCg signals reflect stronger effort discounting for other. We find this possibility less likely as ACCg processes effort both when making a choice and when exerting the force, and people who had stronger effort representation at choice subsequently exerted more force. Interpreting ACCg signals as being important for motivating actions that benefit others, rather than inhibiting them, may explain why lesions to the ACCg impair the effortful process of learning of new prosocial action-outcome associations, but not the execution of low effort previously learned prosocial acts.[76,77] Moreover, our results suggest that the finding of functional connectivity in local field potentials between the amygdala and ACCg when monkeys allocate rewards to others[78] may be linked to

CellPress
OPEN ACCESS

ensuring the monkeys overcome any costs associated with being prosocial. Nevertheless, future work could seek to test these competing hypotheses. Relatedly, future work can examine the domain generality versus domain specificity of ACCg in social behavior. In our paradigm, we independently manipulated the reward and effort costs, but in everyday life, we often have to weigh other costs such as sacrificing time to help. Due to the complexity of our protocol and richness of experimental conditions, it was not possible to compare effort costs with these other costs. Designs that manipulate time and effort costs in the same paradigm could be fruitful for uncovering the precise role of ACCg in social behavior.

The notion that prosocial acts can be considered goal-directed acts, governed by similar principles and computational mechanisms as non-goal-directed actions but implemented in distinct neural regions, highlights the need to examine effort processing to understand what makes people help others.[6] Recently, it has been shown that higher levels of affective empathy are linked to a greater willingness to exert effort to help.[34,79] Here, we show that such individual variability may be linked to ACCg prosocial effort processing, with levels of affective empathy correlated with the strength of multivariate representations of prosocial effort costs when deciding whether to help. Although the link between empathy and prosocial behavior is often discussed, many of the mechanisms providing this link are unclear.[37,79,80] Our findings demonstrate that representing how costly and effortful a prosocial behavior is may be linked to how strongly one represents the emotional states of others, which leads to variability in how willing people are to help others.

Although we found that some regions processed information differently when making decisions about whether to exert effort to benefit self or other, several regions—particularly, the AI and dACC/dmPFC—encoded information, regardless of the beneficiary. Although there has been some debate surrounding whether these regions encode SV in univariate fMRI studies of effort-based decision-making,[18,26,30,31,33,81,82] a large body of evidence suggests that lesions to these regions reduces levels of motivation.[24,32] Neurons in the dACC/dmPFC signal reward value, effort costs, and social identity information.[83,84] In addition, recent meta-analyses of fMRI studies highlights consistent evidence that these regions signal the SV during effort-based decision-making.[24,28] Responses in these areas may be domain general, with SV encoded, regardless of the nature of the effort, whether it is physical or cognitive.[18,28] We show multivariate representations of SV are present in these same regions, regardless of whom the effort is being exerted for. Such a finding supports the idea that neural processes in dACC/dmPFC and AI are an important component of motivated behavior across multiple domains.

Notably, we also found a midbrain region, closely approximating the VTA, that contained a multivariate representation of SV exclusively when making choices to benefit oneself. Previous work across species has linked the VTA to exerting effort for one's own rewards.[81,85–88] Neurophysiological recordings highlight that local field potentials are sensitive to effort requirements and that neurons firing increases prior to deciding whether to exert effort.[10,85] Neuroimaging studies suggest the VTA may be important for learning how to avoid effort costs and when deciding how much effort to allocate to a trial of a task.[81,87,88] However, our results suggest that this region may not process information for all efforts, only for those that benefit oneself. Such findings concord with the idea that prosocial and self-benefiting actions are distinct, may be linked to partially distinct motivational processes, and suggest that perhaps "warm glow" is not always the driver of prosocial acts.[8,34,35,79,80]

Recently, we highlighted how using the framework of Marr's three levels can be fruitful for examining if a process is socially, or self, specific, either in how it is implemented in the brain, or in its algorithmic processes.[12] In line with this approach, our findings highlight the critical importance of breaking prosocial behavior down into its constituent parts, for using multivariate approaches and for designing paradigms that separate self-benefiting from other-benefiting decision-making. Previous work examining prosocial behavior, particularly using economic games, has been crucial for implicating the neural systems.[1,89–91] However, the precise computations have been hard to identify due to the challenge of untangling self-from other-benefiting components. We reveal that several regions, including in the AI and dACC/dmPFC which have been implicated in prosocial behaviors,[49] in fact carry information when making both self-benefiting and other-benefiting choices. This finding raises the possibility that these areas may be less directly linked to prosocial behavior and more linked to domain-general decision processes. In the real world outside of the lab, prosocial behaviors also often occur where people get direct feedback from others. However, many prosocial decisions also occur when dynamic interactions do not feature. Examples include the acts of donating blood, sharing code so that others will benefit, or recycling waste to prevent global warming. It is an important question for future studies to examine how neural signals are modulated by different social contexts, and whether the same or additional brain areas are recruited. In addition, there could be different neural signals that occur between valuing options when offered and when choosing to select them. Future work could attempt to dissociate how value signals unfold, using imaging methods well suited for capturing timing, such as RSA applied to magnetoencephalography data.[92]

In conclusion, many prosocial acts require effort. We find evidence of distinct neural patterns of effort for prosocial and self-benefiting acts. The ACCg carries a multivariate representation of effort when making prosocial choices and is engaged when energizing prosocial acts but does not carry similar self-benefiting information. The AI and dACC/dmPFC track both self-benefiting and prosocial behaviors. In contrast, the VTA processes the structure of subjective value only of self-benefiting acts and the ventral AI more closely tracks self-benefiting, compared with other-benefiting, values. These findings provide new insights into how the brain makes decisions about whether to put in effort to help others out, with important implications for everyday prosocial acts and enhancing them in health and disease.

STAR★METHODS

Detailed methods are provided in the online version of this paper and include the following:

- KEY RESOURCES TABLE
- RESOURCE AVAILABILITY
  - Lead contact
  - Materials availability
  - Data and code availability
- EXPERIMENTAL MODEL AND SUBJECT DETAILS
  - Participants
- METHOD DETAILS
  - Procedure
  - Role assignment
- QUANTIFICATION AND STATISTICAL ANALYSIS
  - Statistical analysis of behavioral data
  - Questionnaire of cognitive and affective empathy
  - Computational modelling of behavioral data
  - Parameter recovery
  - Model identifiability
  - Imaging methods
  - Imaging pre-processing and analyses
  - Imaging design: multivariate analysis
  - Imaging design: Univariate analysis
  - Exploratory ROIs in vmPFC and VS
  - Connectivity analysis

SUPPLEMENTAL INFORMATION

Supplemental information can be found online at https://doi.org/10.1016/j.cub.2022.08.010.

ACKNOWLEDGMENTS

This work was supported by a Medical Research Council Fellowship (MR/P014097/1 and MR/P014097/2), a Sir Henry Dale Fellowship funded by the Wellcome Trust and the Royal Society (223264/Z/21/Z), a Christ Church Junior Research Fellowship, a Christ Church Research Centre Grant, and a Jacobs Foundation Research Fellowship to P.L.L.; a Biotechnology and Biological Sciences Research Council David Phillips Fellowship (BB/R010668/1) and a Wellcome Trust Institutional Strategic Support Fund grant awarded to M.A.J.A.; a Wellcome Trust Principal Fellowship to M.H.; and the National Institute for Health Research Biomedical Research Centre, Oxford, United Kingdom. The Wellcome Centre for Integrative Neuroimaging is supported by core funding from the Wellcome Trust (203139/Z/16/Z). We are grateful to Matthew Rushworth, Miriam Klein-Flugge, and Ali Mahmoodi for helpful discussions and to Tanja Muller for input with experimental code. We are also grateful to our colleagues who acted as the other participant during the study.

AUTHOR CONTRIBUTIONS

Conceptualization, P.L.L. and M.A.J.A.; methodology, P.L.L., M.K.W., H.N., and M.A.J.A.; investigation, P.L.L., M.M.-R., and A.A.; formal analysis, P.L.L., M.K.W., H.N., J.C., and M.A.J.A.; writing – original draft, P.L.L., M.M.-R., and M.A.J.A.; writing – review & editing, P.L.L., M.K.W., H.N., M.M.-R., A.A., J.C., M.H., and M.A.J.A.; funding acquisition, P.L.L., M.A.J.A., and M.H.; supervision, P.L.L., M.A.J.A., and M.H.

DECLARATION OF INTERESTS

The authors declare no competing interests.

INCLUSION AND DIVERSITY

We worked to ensure gender balance in the recruitment of human subjects. We worked to ensure that the study questionnaires were prepared in an inclusive way. One or more of the authors of this paper self-identifies as an underrepresented ethnic minority in science. One or more of the authors of this paper self-identifies as living with a disability. The author list of this paper includes contributors from the location where the research was conducted who participated in the data collection, design, analysis, and/or interpretation of the work.

Received: April 22, 2022
Revised: July 13, 2022
Accepted: August 7, 2022
Published: August 26, 2022

REFERENCES

1. Camerer, C.F. (1997). Progress in behavioral game theory. J. Econ. Perspect. *11*, 167–188.
2. Fehr, E., and Fischbacher, U. (2004). Social norms and human cooperation. Trends Cogn. Sci. *8*, 185–190.
3. Post, S.G. (2005). Altuism, happiness, and health: it's good to be good. Int. J. Behav. Med. *12*, 66–77.
4. Raposa, E.B., Laws, H.B., and Ansell, E.B. (2016). Prosocial behavior mitigates the negative effects of stress in everyday life. Clin. Psychol. Sci. *4*, 691–698.
5. Kosse, F., and Tincani, M.M. (2020). Prosociality predicts labor market success around the world. Nat. Commun. *11*, 5298.
6. Contreras-Huerta, L.S., Pisauro, M.A., and Apps, M.A.J. (2020). Effort shapes social cognition and behaviour: a neuro-cognitive framework. Neurosci. Biobehav. Rev. *118*, 426–439.
7. Hare, T.A., Camerer, C.F., Knoepfle, D.T., and Rangel, A. (2010). Value computations in ventral medial prefrontal cortex during charitable decision making incorporate input from regions involved in social cognition. J. Neurosci. *30*, 583–590.
8. Imas, A. (2014). Working for the "warm glow": on the benefits and limits of prosocial incentives. J. Public Econ. *114*, 14–18.
9. Lockwood, P.L., Hamonet, M., Zhang, S.H., Ratnavel, A., Salmony, F.U., Husain, M., and Apps, M.A.J. (2017). Prosocial apathy for helping others when effort is required. Nat. Hum. Behav. *1*, 0131.
10. Varazzani, C., San-Galli, A., Gilardeau, S., and Bouret, S. (2015). Noradrenaline and dopamine neurons in the reward/effort trade-off: a direct electrophysiological comparison in behaving monkeys. J. Neurosci. *35*, 7866–7877.
11. Krakauer, J.W., Ghazanfar, A.A., Gomez-Marin, A., MacIver, M.A., and Poeppel, D. (2017). Neuroscience needs behavior: correcting a reductionist bias. Neuron *93*, 480–490.
12. Lockwood, P.L., Apps, M.A.J., and Chang, S.W.C. (2020). Is there a 'social' brain? Implementations and algorithms. Trends Cogn. Sci. *24*, 802–813.
13. Niv, Y. (2021). The primacy of behavioral research for understanding the brain. Behav. Neurosci. *135*, 601–609.
14. Apps, M.A., Grima, L.L., Manohar, S., and Husain, M. (2015). The role of cognitive effort in subjective reward devaluation and risky decision-making. Sci. Rep. *5*, 16880.
15. Hull, C.L. (1943). Principles of Behavior: An Introduction to Behavior Theory (Appleton-Century).
16. Kool, W., and Botvinick, M. (2018). Mental labour. Nat. Hum. Behav. *2*, 899–908.
17. Westbrook, A., and Braver, T.S. (2015). Cognitive effort: a neuroeconomic approach. Cogn. Affect. Behav. Neurosci. *15*, 395–415.
18. Chong, T.T.-J., Apps, M., Giehl, K., Sillence, A., Grima, L.L., and Husain, M. (2017). Neurocomputational mechanisms underlying subjective valuation of effort costs. PLoS Biol. *15*, e1002598.
19. Hartmann, M.N., Hager, O.M., Tobler, P.N., and Kaiser, S. (2013). Parabolic discounting of monetary rewards by physical effort. Behav. Processes *100*, 192–196.
20. Klein-Flügge, M.C., Kennerley, S.W., Saraiva, A.C., Penny, W.D., and Bestmann, S. (2015). Behavioral modeling of human choices reveals

CellPress
OPEN ACCESS

dissociable effects of physical effort and temporal delay on reward devaluation. PLoS Comput. Biol. *11*, e1004116.

21. Hart, E.E., Gerson, J.O., Zoken, Y., Garcia, M., and Izquierdo, A. (2017). Anterior cingulate cortex supports effort allocation towards a qualitatively preferred option. Eur. J. Neurosci. *46*, 1682–1688.
22. Hart, E.E., Blair, G.J., O'Dell, T.J., Blair, H.T., and Izquierdo, A. (2020). Chemogenetic modulation and single-photon calcium imaging in anterior cingulate cortex reveal a mechanism for effort-based decisions. J. Neurosci. *40*, 5628–5643.
23. Hu, Y., van Wingerden, M., Sellitto, M., Schäble, S., and Kalenscher, T. (2021). Anterior cingulate cortex lesions abolish budget effects on effort-based decision-making in rat consumers. J. Neurosci. *41*, 4448–4460.
24. Pessiglione, M., Vinckier, F., Bouret, S., Daunizeau, J., and Le Bouc, R. (2018). Why not try harder? Computational approach to motivation deficits in neuro-psychiatric diseases. Brain *141*, 629–650.
25. Walton, M.E., Kennerley, S.W., Bannerman, D.M., Phillips, P.E.M., and Rushworth, M.F.S. (2006). Weighing up the benefits of work: behavioral and neural analyses of effort-related decision making. Neural Netw. *19*, 1302–1314.
26. Croxson, P.L., Walton, M.E., O'Reilly, J.X., Behrens, T.E.J., and Rushworth, M.F.S. (2009). Effort-based cost-benefit valuation and the human brain. J. Neurosci. *29*, 4531–4541.
27. Holroyd, C.B., and McClure, S.M. (2015). Hierarchical control over effortful behavior by rodent medial frontal cortex: a computational model. Psychol. Rev. *122*, 54–83.
28. Lopez-Gamundi, P., Yao, Y.-W., Chong, T.T., Heekeren, H.R., Herrero, E.M., and Pallares, J.M. (2021). The neural basis of effort valuation: a meta-analysis of functional magnetic resonance imaging studies. Neurosci. Biobehav. Rev. *131*, 1275–1287.
29. Soutschek, A., Kang, P., Ruff, C.C., Hare, T.A., and Tobler, P.N. (2018). Brain stimulation over the frontopolar cortex enhances motivation to exert effort for reward. Biol. Psychiatry *84*, 38–45.
30. Vassena, E., Silvetti, M., Boehler, C.N., Achten, E., Fias, W., and Verguts, T. (2014). Overlapping neural systems represent cognitive effort and reward anticipation. PLoS One *9*, e91008.
31. Verguts, T., Vassena, E., and Silvetti, M. (2015). Adaptive effort investment in cognitive and physical tasks: a neurocomputational model. Front. Behav. Neurosci. *9*, 57.
32. Le Heron, C., Apps, M.A.J., and Husain, M. (2018). The anatomy of apathy: a neurocognitive framework for amotivated behavior. Neuropsychologia *118*, 54–67.
33. Müller, T., Klein-Flügge, M.C., Manohar, S.G., Husain, M., and Apps, M.A.J. (2021). Neural and computational mechanisms of momentary fatigue and persistence in effort-based choice. Nat. Commun. *12*, 4593.
34. Contreras-Huerta, L.S., Lockwood, P.L., Bird, G., Apps, M.A.J., and Crockett, M.J. (2022). Prosocial behavior is associated with transdiagnostic markers of affective sensitivity in multiple domains. Emotion *22*, 820–835.
35. Lockwood, P.L., Abdurahman, A., Gabay, A.S., Drew, D., Tamm, M., Husain, M., and Apps, M.A.J. (2021). Aging increases prosocial motivation for effort. Psychol. Sci. *32*, 668–681.
36. Apps, M.A.J., Rushworth, M.F.S., and Chang, S.W.C. (2016). The anterior cingulate gyrus and social cognition: tracking the motivation of others. Neuron *90*, 692–707.
37. Lockwood, P.L. (2016). The anatomy of empathy: vicarious experience and disorders of social cognition. Behav. Brain Res. *311*, 255–266.
38. Chang, S.W.C., Gariépy, J.-F., and Platt, M.L. (2013). Neuronal reference frames for social decisions in primate frontal cortex. Nat. Neurosci. *16*, 243–250.
39. Apps, M.A.J., and Ramnani, N. (2014). The anterior cingulate gyrus signals the net value of others' rewards. J. Neurosci. *34*, 6190–6200.
40. Apps, M.A., Lesage, E., and Ramnani, N. (2015). Vicarious reinforcement learning signals when instructing others. J. Neurosci. *35*, 2904–2913.
41. Lockwood, P.L., Apps, M.A., Roiser, J.P., and Viding, E. (2015). Encoding of vicarious reward prediction in anterior cingulate cortex and relationship with trait empathy. J. Neurosci. *35*, 13720–13727.
42. Lockwood, P.L., Wittmann, M.K., Apps, M.A.J., Klein-Flügge, M.C., Crockett, M.J., Humphreys, G.W., and Rushworth, M.F.S. (2018). Neural mechanisms for learning self and other ownership. Nat. Commun. *9*, 4747.
43. Lamm, C., Rütgen, M., and Wagner, I.C. (2019). Imaging empathy and prosocial emotions. Neurosci. Lett. *693*, 49–53.
44. Zaki, J. (2014). Empathy: a motivated account. Psychol. Bull. *140*, 1608–1647.
45. Crockett, M.J., Siegel, J.Z., Kurth-Nelson, Z., Dayan, P., and Dolan, R.J. (2017). Moral transgressions corrupt neural representations of value. Nat. Neurosci. *20*, 879–885.
46. Le Bouc, R., and Pessiglione, M. (2013). Imaging social motivation: distinct brain mechanisms drive effort production during collaboration versus competition. J. Neurosci. *33*, 15894–15902.
47. Morishima, Y., Schunk, D., Bruhin, A., Ruff, C.C., and Fehr, E. (2012). Linking brain structure and activation in temporoparietal junction to explain the neurobiology of human altruism. Neuron *75*, 73–79.
48. Obeso, I., Moisa, M., Ruff, C.C., and Dreher, J.-C. (2018). A causal role for right temporo-parietal junction in signaling moral conflict. eLife *7*, e40671.
49. Ruff, C.C., and Fehr, E. (2014). The neurobiology of rewards and values in social decision making. Nat. Rev. Neurosci. *15*, 549–562.
50. Seltzer, B., and Pandya, D.N. (1989). Frontal lobe connections of the superior temporal sulcus in the rhesus monkey. J. Comp. Neurol. *281*, 97–113.
51. Vogt, B.A., and Pandya, D.N. (1987). Cingulate cortex of the rhesus monkey: 2. Cortical afferents. J. Comp. Neurol. *262*, 271–289.
52. Averbeck, B.B., Latham, P.E., and Pouget, A. (2006). Neural correlations, population coding and computation. Nat. Rev. Neurosci. *7*, 358–366.
53. Kriegeskorte, N., and Kievit, R.A. (2013). Representational geometry: integrating cognition, computation, and the brain. Trends Cogn. Sci. *17*, 401–412.
54. Palminteri, S., Wyart, V., and Koechlin, E. (2017). The importance of falsification in computational cognitive modeling. Trends Cogn. Sci. *21*, 425–433.
55. Lockwood, P.L., and Klein-Flügge, M.C. (2021). Computational modelling of social cognition and behaviour—a reinforcement learning primer. Soc. Cogn. Affect. Neurosci. *16*, 761–771.
56. Nili, H., Wingfield, C., Walther, A., Su, L., Marslen-Wilson, W., and Kriegeskorte, N. (2014). A toolbox for representational similarity analysis. PLoS Comput. Biol. *10*, e1003553.
57. Love, B.C. (2015). The algorithmic level is the bridge between computation and brain. Top. Cogn. Sci. *7*, 230–242.
58. Dimsdale-Zucker, H.R., and Ranganath, C. (2018). Representational similarity analyses. Handbook of Behavioral Neuroscience (Elsevier), pp. 509–525.
59. Kriegeskorte, N., Mur, M., and Bandettini, P.A. (2008). Representational similarity analysis - connecting the branches of systems neuroscience. Front. Syst. Neurosci. *2*, 4.
60. Popal, H., Wang, Y., and Olson, I.R. (2019). A guide to representational similarity analysis for social neuroscience. Soc. Cogn. Affect. Neurosci. *14*, 1243–1253.
61. Wittmann, M.K., Lockwood, P.L., and Rushworth, M.F.S. (2018). Neural mechanisms of social cognition in primates. Annu. Rev. Neurosci. *41*, 99–118.
62. Neubert, F.-X., Mars, R.B., Sallet, J., and Rushworth, M.F.S. (2015). Connectivity reveals relationship of brain areas for reward-guided

learning and decision making in human and monkey frontal cortex. Proc. Natl. Acad. Sci. USA *112*, E2695–E2704.

63. Reniers, R.L., Corcoran, R., Drake, R., Shryane, N.M., and Völlm, B.A. (2011). The QCAE: a questionnaire of cognitive and affective empathy. J. Pers. Assess. *93*, 84–95.
64. Basti, A., Nili, H., Hauk, O., Marzetti, L., and Henson, R.N. (2020). Multi-dimensional connectivity: a conceptual and mathematical review. NeuroImage *221*, 117179.
65. Lockwood, P.L., Apps, M.A.J., Valton, V., Viding, E., and Roiser, J.P. (2016). Neurocomputational mechanisms of prosocial learning and links to empathy. Proc. Natl. Acad. Sci. USA *113*, 9763–9768.
66. Decety, J., and Ickes, W. (2011). The Social Neuroscience of Empathy (MIT Press).
67. Singer, T., and Lamm, C. (2009). The social neuroscience of empathy. Ann. NY Acad. Sci. *1156*, 81–96.
68. Singer, T., Seymour, B., O'Doherty, J., Kaube, H., Dolan, R.J., and Frith, C.D. (2004). Empathy for pain involves the affective but not sensory components of pain. Science *303*, 1157–1162.
69. Lebreton, M., Bavard, S., Daunizeau, J., and Palminteri, S. (2019). Assessing inter-individual differences with task-related functional neuroimaging. Nat. Hum. Behav. *3*, 897–905.
70. Walther, A., Nili, H., Ejaz, N., Alink, A., Kriegeskorte, N., and Diedrichsen, J. (2016). Reliability of dissimilarity measures for multi-voxel pattern analysis. NeuroImage *137*, 188–200.
71. Rudebeck, P.H., Buckley, M.J., Walton, M.E., and Rushworth, M.F.S. (2006). A role for the macaque anterior cingulate gyrus in social valuation. Science *313*, 1310–1312.
72. Carrillo, M., Han, Y., Migliorati, F., Liu, M., Gazzola, V., and Keysers, C. (2019). Emotional mirror neurons in the Rat's anterior cingulate cortex. Curr. Biol. *29*, 1301–1312.e6.
73. Han, Y., Bruls, R., Soyman, E., Thomas, R.M., Pentaraki, V., Jelinek, N., Heinemans, M., Bassez, I., Verschooren, S., Pruis, I., et al. (2019). Bidirectional cingulate-dependent danger information transfer across rats. PLoS Biol. *17*, e3000524.
74. Hillman, K.L., and Bilkey, D.K. (2012). Neural encoding of competitive effort in the anterior cingulate cortex. Nat. Neurosci. *15*, 1290–1297.
75. Hill, M.R., Boorman, E.D., and Fried, I. (2016). Observational learning computations in neurons of the human anterior cingulate cortex. Nat. Commun. *7*, 12722.
76. Basile, B.M., Schafroth, J.L., Karaskiewicz, C.L., Chang, S.W.C., and Murray, E.A. (2020). The anterior cingulate cortex is necessary for forming prosocial preferences from vicarious reinforcement in monkeys. PLoS Biol. *18*, e3000677.
77. Lockwood, P.L., O'Nell, K.C., and Apps, M.A.J. (2020). Anterior cingulate cortex: a brain system necessary for learning to reward others? PLoS Biol. *18*, e3000735.
78. Dal Monte, O., Chu, C.C.J., Fagan, N.A., and Chang, S.W.C. (2020). Specialized medial prefrontal–amygdala coordination in other-regarding decision preference. Nat. Neurosci. *23*, 565–574.
79. Lockwood, P.L., Ang, Y.-S., Husain, M., and Crockett, M.J. (2017). Individual differences in empathy are associated with apathy-motivation. Sci. Rep. *7*, 17293.
80. Morelli, S.A., Sacchet, M.D., and Zaki, J. (2015). Common and distinct neural correlates of personal and vicarious reward: a quantitative meta-analysis. NeuroImage *112*, 244–253.
81. Hauser, T.U., Eldar, E., and Dolan, R.J. (2017). Separate mesocortical and mesolimbic pathways encode effort and reward learning signals. Proc. Natl. Acad. Sci. USA *114*, E7395–E7404.
82. Westbrook, A., Lamichhane, B., and Braver, T. (2019). The subjective value of cognitive effort is encoded by a domain-general valuation network. J. Neurosci. *39*, 3934–3947.
83. Kennerley, S.W., Dahmubed, A.F., Lara, A.H., and Wallis, J.D. (2009). Neurons in the frontal lobe encode the value of multiple decision variables. J. Cogn. Neurosci. *21*, 1162–1178.
84. Báez-Mendoza, R., Mastrobattista, E.P., Wang, A.J., and Williams, Z.M. (2021). Social agent identity cells in the prefrontal cortex of interacting groups of primates. Science *374*, eabb4149.
85. Elston, T.W., and Bilkey, D.K. (2017). Anterior cingulate cortex modulation of the ventral tegmental area in an effort task. Cell Rep. *19*, 2220–2230.
86. Hamid, A.A., Pettibone, J.R., Mabrouk, O.S., Hetrick, V.L., Schmidt, R., Vander Weele, C.M., Kennedy, R.T., Aragona, B.J., and Berke, J.D. (2016). Mesolimbic dopamine signals the value of work. Nat. Neurosci. *19*, 117–126.
87. Kroemer, N.B., Guevara, A., Ciocanea Teodorescu, I.C., Wuttig, F., Kobiella, A., and Smolka, M.N. (2014). Balancing reward and work: anticipatory brain activation in NAcc and VTA predict effort differentially. Neuroimage *102*, 510–519.
88. Kurniawan, I.T., Guitart-Masip, M., Dayan, P., and Dolan, R.J. (2013). Effort and valuation in the brain: the effects of anticipation and execution. J. Neurosci. *33*, 6160–6169.
89. Fehr, E., and Camerer, C.F. (2007). Social neuroeconomics: the neural circuitry of social preferences. Trends Cogn. Sci. *11*, 419–427.
90. King-Casas, B., Tomlin, D., Anen, C., Camerer, C.F., Quartz, S.R., and Montague, P.R. (2005). Getting to know you: reputation and trust in a two-person economic exchange. Science *308*, 78–83.
91. Tomlin, D., Kayali, M.A., King-Casas, B., Anen, C., Camerer, C.F., Quartz, S.R., and Montague, P.R. (2006). Agent-specific responses in the cingulate cortex during economic exchanges. Science *312*, 1047–1050.
92. Cichy, R.M., and Oliva, A. (2020). A M/EEG-fMRI fusion primer: resolving human brain responses in space and time. Neuron *107*, 772–781.
93. Crockett, M.J., Kurth-Nelson, Z., Siegel, J.Z., Dayan, P., and Dolan, R.J. (2015). Harm to others outweighs harm to self in moral decision making. Proc. Natl. Acad. Sci. USA *112*, 17320–17325.
94. Fehr, E., and Schmidt, K.M. (2006). Chapter 8. The economics of fairness, reciprocity and altruism – experimental evidence and new theories. In Handbook of the Economics of Giving, Altruism and Reciprocity Foundations, S.-C. Kolm, and J.M. Ythier, eds. (Elsevier), pp. 615–691.
95. Lockwood, P.L., Klein-Flügge, M.C., Abdurahman, A., and Crockett, M.J. (2020). Model-free decision making is prioritized when learning to avoid harming others. Proc. Natl. Acad. Sci. USA *117*, 27719–27730.
96. R Core Team (2017). R: a language and environment for statistical computing (R Foundation for Statistical Computing).
97. RStudio Team (2015). RStudio: Integrated Development for R. (R Foundation for Statistical Computing) https://www.rstudio.com/categories/integrated-development-environment/.
98. Barr, D.J., Levy, R., Scheepers, C., and Tily, H.J. (2013). Random effects structure for confirmatory hypothesis testing: keep it maximal. J. Mem. Lang. *68*, 255–278.
99. Singmann, H., Bolker, B., Westfall, J., Aust, F., and Ben-Shachar, M.S. (2019). Afex: analysis of factorial experiments. R Package Version 0.25-1.
100. Bates, D., Mächler, M., Bolker, B., and Walker, S. (2015). Fitting linear mixed-effects models using lme4. J. Stat. Softw. *67*, 1–48.
101. Kassambara, A. (2020). Rstatix: pipe-friendly framework for basic statistical tests. R Package Version 0.5.0.
102. Revelle, W., and Revelle, M.W. (2015). Package 'Psych'. Compr. R Arch. Netw.
103. Deichmann, R., Gottfried, J.A., Hutton, C., and Turner, R. (2003). Optimized EPI for fMRI studies of the orbitofrontal cortex. Neuroimage *19*, 430–441.
104. Ashburner, J., and Friston, K.J. (2005). Unified segmentation. NeuroImage *26*, 839–851.

105. Hendriks, M.H.A., Daniels, N., Pegado, F., and Op de Beeck, H.P. (2017). The effect of spatial smoothing on representational similarity in a simple motor paradigm. Front. Neurol. *8*, 222.

106. Bang, D., Ershadmanesh, S., Nili, H., and Fleming, S.M. (2020). Private-public mappings in human prefrontal cortex. eLife *9*, e56477.

107. Baram, A.B., Muller, T.H., Nili, H., Garvert, M.M., and Behrens, T.E.J. (2021). Entorhinal and ventromedial prefrontal cortices abstract and generalize the structure of reinforcement learning problems. Neuron *109*, 713–723.e7.

108. Park, S.A., Miller, D.S., Nili, H., Ranganath, C., and Boorman, E.D. (2020). Map making: constructing, combining, and inferring on abstract cognitive maps. Neuron *107*, 1226–1238.e8.

109. Eklund, A., Nichols, T.E., and Knutsson, H. (2016). Cluster failure: why fMRI inferences for spatial extent have inflated false-positive rates. Proc. Natl. Acad. Sci. USA *113*, 7900–7905.

110. Hall-McMaster, S., Muhle-Karbe, P.S., Myers, N.E., and Stokes, M.G. (2019). Reward boosts neural coding of task rules to optimize cognitive flexibility. J. Neurosci. *39*, 8549–8561.

111. Penny, W.D., Friston, K.J., Ashburner, J.T., Kiebel, S.J., and Nichols, T.E. (2011). Statistical Parametric Mapping: The Analysis of Functional Brain Images (Elsevier).

112. Will, G.J., Rutledge, R.B., Moutoussis, M., and Dolan, R.J. (2017). Neural and computational processes underlying dynamic changes in self-esteem. eLife *6*, e28098.

113. Slotnick, S.D. (2017). Cluster success: fMRI inferences for spatial extent have acceptable false-positive rates. Cogn. Neurosci. *8*, 150–155.

114. Flandin, G., and Friston, K.J. (2019). Analysis of family-wise error rates in statistical parametric mapping using random field theory. Brain Mapp. *40*, 2052–2054.

STAR★METHODS

KEY RESOURCES TABLE

REAGENT or RESOURCE	SOURCE	IDENTIFIER
Software and algorithms		
Matlab v2019b	Mathworks	https://www.mathworks.com
SPM12	UCL, UK	https://www.fil.ion.ucl.ac.uk/spm/software/
Psychtoolbox3	Psychtoolbox	http://psychtoolbox.org/
RSA toolbox	Nili et al.[56]	https://git.fmrib.ox.ac.uk/hnili/rsa
Acqknowledge	BIOPAC Systems UK	https://www.biopac.com/product/acqknowledge-software/
R	The R Foundation	N/A
Other		
Hand clench Dynamometer for MRI (TSD121B-MRI)	BIOPAC	https://www.biopac.com/product/hand-clench-dynamom-for-mri/

RESOURCE AVAILABILITY

Lead contact

Further information and requests for resources and reagents should be directed to and will be fulfilled by the Lead Contact, Patricia L. Lockwood (p.l.lockwood@bham.ac.uk).

Materials availability

This study neither used any reagent nor generated new materials.

Data and code availability

- All anonymized behavioral data and code used to generate the figures can be downloaded at OSF (https://osf.io/tm45q).
- All code used to run the computational modelling can be downloaded at OSF (https://osf.io/tm45q). Unthresholded statistical maps can be downloaded at NeuroVault (https://identifiers.org/neurovault.collection:12789).

EXPERIMENTAL MODEL AND SUBJECT DETAILS

Participants

41 healthy, right-handed participants took part. Our pre-scanning exclusion criteria were previous psychology experience, participation in social studies, left handedness, and neuro/psychiatric disorders. These questions were asked via an online screening procedure and only participants who met these criteria were invited to take part. Our post-scanning exclusion criteria were disbelief in the deception or lack of selecting the work option on any trial for self and other. Three participants were excluded based on this post-scanning exclusion criteria. Two who did not believe the deception in the study set-up (see "Role assignment" details below), and one who never chose to exert effort for the other person. The final group of 38 participants (26 females, mean age 23, range 18-34). Based on the effect size from Lockwood et al.,[9] a sample of 38 people gave 83% power to detect a significant behavioral effect.

Participants were recruited through student mailing lists, online advertisements on a study recruitment board, through social media, and by word of mouth. The study was described as a social decision-making study involving pairs of participants. Participants believed that, on the day of testing, one of the pair would be randomly allocated to complete the task in the fMRI scanner whilst the other would complete the task in a testing room. In reality, all participants completed the task in the scanner, and a confederate served as the other participant. The study was approved by the Medical Sciences Division Research Ethics Committee of the University of Oxford. All participants provided written informed consent. Participants were paid for their participation at a rate of £15/hour, plus a bonus of up to £5 based on the credits they earned in the task. They were also told the number of credits that they earned in the prosocial condition would translate into an additional payment of up to £5 for the other participant (see details of the task below).

METHOD DETAILS

Procedure

Approximately 1 week before attending the testing session, participants completed a questionnaire assessment of empathy online using the Questionnaire of Cognitive and Affective Empathy (see below for further details). Participants then attended the lab to

complete a physical effort-based decision-making task modified for scanning from previous behavioral studies.[9,35] Physical effort was operationalized as the amount of force participants exerted on a handheld dynamometer. On arrival and after consent, participants were instructed to squeeze the handheld dynamometer as hard as they could. Participants were provided with visual feedback whilst doing so and encouraged to reach a line that was 110% of their maximum voluntary contraction (MVC) which they repeated for 3 trials. After this thresholding procedure, and before any task instructions, participants were introduced to another participant anonymously (see "role assignment' procedure below). Participants practiced each of the 5 effort levels twice to ensure that they could be achieved. In the main task inside the fMRI scanner, participants were prompted to choose between one of two offers on each trial. One option allowed participants to earn a low reward for low effort (rest); the other presented a variable higher-reward, higher-effort offer (work) of the same duration. The low-reward, low-effort offer earned 1 point and required no effort. Higher-reward, higher-effort offers varied from 2-10 points (in 2-point increments). Effort ranged from 30-70% (in 10% increments) of the participants' MVC. Participants were instructed that they could win a bonus of up to £5 and that more points earned corresponded to a greater bonus, but were not made aware of the exchange rate while completing the task to ensure that they did not try to compute a running total. Critically, each trial also varied in whether the outcome would be delivered the participant themselves (Self) or the receiver participant (Other, prosocial). The level of effort required for each offer was represented using colored portions of a pie chart (Figure 1A). Rewards (points) on offer for each option were written in color below. Participants were allotted 3.5 seconds to make a choice between the rest and work offers. If they failed to choose an option, they were awarded 0 points after a full trial duration. After choosing, participants were shown a screen with a yellow horizontal bar on an empty vertical box. The horizontal bar represented the level of effort required; the box filled according to the force participants exerted on the dynamometer, providing feedback in real-time. For a trial to be considered successful, and rewards obtained, participants had to accumulate at least 1 second at or above the required force level across the 3 second force period.

The task was broken into four blocks, with a minute break in between each block to rest and prevent the build-up of fatigue. We also empirically assessed whether failure rates or willingness to accept the high-effort option for higher rewards shifted over the course of the experiment, which could reflect fatigue. Trial number did not have a significant effect in predicting success in meeting the effort requirement (OR=1.00 [0.83, 1.20], $p = 0.98$) or predicting choices to work / rest (OR=0.85 [0.68, 1.06], $p = 0.15$). There was also not an interaction between trial number and recipient for either success rate (OR=0.89 [0.75, 1.07], $p = 0.22$) or choices (OR=0.97 [0.85, 1.11], $p = 0.68$). Participants selected the choice they wanted using a game controller in their left hand and used their right hand to squeeze the dynamometer. Each participant completed 100 interleaved trials per recipient (self or other).

Role assignment

Participants were introduced to another participant who was in fact a confederate of the experimenter, as in previous studies of social decision-making[35,93] (Figure 1B). Participants were instructed not to speak and wore a glove to hide any physical characteristics and to ensure they were anonymous to one another. A second experimenter brought the confederate to the other side of the door who was also instructed not to speak and wore a glove. Participants only ever saw the gloved hand of the confederate, but they waved to each other to make it clear there was another person there (Figure 1B). The experimenter tossed a coin to determine who picked a ball from the box first and then told the participants which roles they had been assigned to, based on the ball that they picked. Unbeknownst to participants, our procedure ensured that participants always ended up in the role of the person performing the effort task inside the MRI scanner and they were led to believe the other participant would be performing tasks outside of the scanner. We emphasized that the participant outside of the scanner would only perform experimental tasks that would result in outcomes for themselves and would be unaware of the task performed by the participant inside the scanner, so any reward given would be anonymous. This procedure minimized as much as possible any prosocial behavior being due to social preferences of reciprocity.[94] We revealed the first name of the other participant, that was always gender matched to the participant performing the experiment, to further emphasize the recipient of rewards on 'other' trials.

After finishing the task in the scanner, participants completed a short debriefing questionnaire where they were probed as to whether they believed they were earning rewards for another participant. Two participants reported a disbelief in the deception and were removed from analysis. We excluded these participants (in line with our previous work using this role-assignment procedure[9,95]) as their behavior on the task and associated neural processes would not reflect decisions to help, or fail to help, another person, since these participants did not truly believe that their decisions would have any influence on the outcomes for someone else.

QUANTIFICATION AND STATISTICAL ANALYSIS

Statistical analysis of behavioral data

Analyses of behavioral data were performed using a combination of MATLAB (2019, The MathWorks) and R (version 3.6.2) using RStudio.[96,97] For choices between the work and rest offers we coded choice as a binary outcome variable and ran a generalized linear mixed-effects model (GLMM). The maximal possible model included fixed and random effects of recipient, effort (squared to mirror the winning parabolic computational model), reward, and all interactions, plus a subject-level random intercept. Squared effort and reward were *Z* scored before being entered into the model. Neither this maximal GLMM of choices or the maximal LMM of normalized force (see below) converged, even with increased iterations ($2x10^5$) and bobyqa optimizer. We therefore reduced the models in the recommended way,[98] removing correlations and the random terms that did not explain any variance, then report the

maximal converging models. To enable removing correlations for random slopes of factorial predictors, we fit models with the mixed function from the afex package[99] which relies on the glmer function from the lme4 package.[100] The final model of choices contained subject-level random effects were uncorrelated slopes for recipient, effort, and reward, all two-way interactions between these variables, and the intercept. Both the GLMM of choices and LMM of force were fit by maximum likelihood and we tested the fixed effects for statistical significance using parametric bootstrapping (1000 simulations) with the mixed function. We used type II tests meaning the significance of a variable was tested by comparing the full model with the next most complex model that does not include that variable. For completeness, we also report Z statistics for the GLMM of choices and χ^2 statistics for both models from these comparisons (Tables S1 and S2). All factors in these models were coded with sum-to-zero contrasts. We exponentiated the standardized coefficients and standard errors for the GLMM of choices to generate odds ratios and their 95% confidence intervals.

Due to the non-normal distribution of the *K* parameters, we compared discounting for self and other using a non-parametric Wilcoxon two-sided signed rank test and generated a standardized effect size (*r*) for this difference using the *wilcox_effsize* function from the rstatix package.[101] For analysis of force exerted following a choice to work, we normalized participants' force as a proportion of their maximum to account for between-subject variability in force exerted and calculated the area under the curve for the 3-second window in which they exerted force. We then analyzed normalized force using a linear mixed-effects model (LMM), starting with a maximal model that contained fixed and random effects of recipient, effort level, reward level, and all interactions, plus a subject-level random intercept. All aspects of the model fitting, reduction and reporting were as with choices above. The final model of normalized force had fixed effects of recipient, effort level, reward level, and all interactions, plus a subject-level random intercept and uncorrelated random slopes for recipient and effort.

Questionnaire of cognitive and affective empathy

Before the testing session, participants completed an online pre-testing questionnaire. The questionnaire aimed to measure individual levels of empathy that might influence prosocial behavior. Empathy is the ability to vicariously experience and understand the affect of other people.[37,67] This ability modulates people's social behavior and is therefore critical to social cognition and social decision-making. The Questionnaire of Cognitive and Affective Empathy (QCAE) measures two dimensions of empathy cognitive and affective.[63] Items in the QCAE corresponded to measures of cognitive empathy (such as *I can easily work out what another person might want to talk about*) or affective empathy (*I am happy when I am with a cheerful group and sad when the others are glum*). Participants rated how much each item applied to them using a 4-point Likert scale from strongly agree to strongly disagree.[63] We used Pearson correlations to test the link between QCAE scores and multivariate representations of prosocial effort in ACCg and compared correlations using the *paired.r* function from the psych package.[102]

Computational modelling of behavioral data

For modelling of choice behavior using trial-by-trial updates, we evaluated a number of plausible models based on past research on effort discounting.[18–20,35] Models were fitted using maximum likelihood estimation using the MATLAB function fmincon.[9,35] For formal model comparison, we report the Bayesian information criterion (BIC) based on the log-likelihood. The model space tested varied the shape of the discount function (*K*) of subjective value, of choosing the more effortful option over the rest option (see below: either (a) parabolic (models 1,4,7,10), (b) linear (models 2,5,8,11), or (c) hyperbolic (models 3,6,9,12)). We also tested models with single and separate noise (β) parameters and whether the same or a different discount parameter was needed for self and other (models 1-6 vs. 7-12). This resulted in 12 putative models (3 different discount functions, separate or the same discount parameters for self and other, and separate or the same noise parameters for self and other), as in previous work.[4,5] Here, we also considered classes of identical models that included free weights on reward (in addition to effort, models 13-24), free weights on reward only (Models 25–36), and models with a *K* parameter scaling the difference between effort and reward (models 37-40). However, these additional models were either not identifiable (Figure S3) or provided worse fits than our original winning model (model 7, separate *K* parameters, single noise parameter) and were not considered further. The full model space was thus defined as follows:

Model 1: Parabolic, 1K1β
Model 2: Linear, 1K1β
Model 3: Hyperbolic, 1K1β
Model 4: Parabolic, 1K2β
Model 5: Linear, 1K2β
Model 6: Hyperbolic, 1K2β
Model 7: Parabolic, 2K1β
Model 8: Linear, 2K1β
Model 9: Hyperbolic, 2K1β
Model 10: Parabolic, 2K2β
Model 11: Linear, 2K2β
Model 12: Hyperbolic, 2K2β
Model 13: Parabolic, 1r1K1β
Model 14: Linear, 1r1K1β
Model 15: Hyperbolic, 1r1K1β

CellPress
OPEN ACCESS

Model 16: Parabolic, $1r1K2\beta$
Model 17: Linear, $1r1K2\beta$
Model 18: Hyperbolic, $1r1K2\beta$
Model 19: Parabolic, $2r2K1\beta$
Model 20: Linear, $2r2K1\beta$
Model 21: Hyperbolic, $2r2K1\beta$
Model 22: Parabolic, $2r2K2\beta$
Model 23: Linear, $2r2K2\beta$
Model 24: Hyperbolic, $2r2K2\beta$
Model 25: Parabolic, $1r1\beta$
Model 26: Linear, $1r1\beta$
Model 27: Hyperbolic, $1r1\beta$
Model 28: Parabolic, $1r2\beta$
Model 29: Linear, $1r2\beta$
Model 30: Hyperbolic, $1r2\beta$
Model 31: Parabolic, $2r1\beta$
Model 32: Linear, $2r1\beta$
Model 33: Hyperbolic, $2r1\beta$
Model 34: Parabolic, $2r2\beta$
Model 35: Linear, $2r2\beta$
Model 36: Hyperbolic, $2r2\beta$
Model 37: Linear, $1K1\beta$
Model 38: Linear, $1K2\beta$
Model 39: Linear, $2K1\beta$
Model 40: Linear, $2K2\beta$

The linear, hyperbolic and parabolic models were specified as follows:

(a) Parabolic: $(t) = (t)\text{-}E(t)^2)$
(b) Linear: $(t) = (t)\cdot(1 - E(t))$
(c) Hyperbolic: $SV(t) = R(t).\frac{1}{1+E(t)}$

The models assumed that the subjective value (SV) of the offer on trial (t) is determined by the effort level (E) (scaled to the proportion of the MVC) and reward level (R) (the number of credits) and the subject-specific parameter. In models 1-24, the discounting parameter (K), describes the steepness of each individual's devaluation of rewards by effort. Thus, the higher the K value, the steeper the discount function. Other models applied a parameter to reward (r models 13-36) or the difference between reward and effort (models 37-40). Note that each individual's discounting function is referenced to the SV of the baseline offer (which was always 1).

The *softmax* function was defined as:

$$\Pr(i) = \frac{e^{\beta.SV_i}}{e^{\beta} + e^{\beta.SV_i}}$$

where Pr(i) represents the probability of choosing option i that has a subjective value of (i), and β is the *softmax* parameter that defines the stochasticity of each participant's choices.

The winning model (model 7), that explained behavior in the majority of participants, was a parabolic model with separate discount (K) parameters and a single noise (β) parameter. This model was very close in BIC value to another model (model 10) also with separate K parameters, but separate noise parameters (Model 7 $2K1\beta$ BIC=4,7948 vs. Model 10 BIC=4,7732 $2K2\beta$). However, the $2K2\beta$ model only won in 33% of participants. We therefore selected model 7 as the winning model. We also conducted both parameter recovery (Figure 2E) and model identifiability to confirm the robustness of our model (see Figure S3).

All discount parameters (K) were bounded between 0 and 1.5. The bounding was empirically determined to capture the range of possible discount values, based on the subjective value for choosing the work offer over the rest offer, given the available reward and effort levels that comprised each offer and the discount function of the winning $2K1\beta$ model. A discount rate of 0 means that a participant would always choose the work offer over the rest offer, whereas a discount rate of 1.5 would mean the participant never chose the work offer over the rest offer. While values of K between 1.5 and 3 are theoretically possible if the discount function were linear instead of parabolic, fitting the models with K bounded between 0 and 3 did not change the model comparison results (Figure S3D) or K values (correlation of 1.00 between values when $0<K<1.5$ and $0<K<3$).

Parameter recovery

Parameter recovery was performed on data simulated by the winning $2K1\beta$ model from 25,856 synthetic participants. We used a wide range of parameter values from a grid of values in the ranges: K_{self}=[0:0.1:1.5]; K_{other}=[0:0.1:1.5]; β=[0:0.1:10], creating 25,856 combinations. We added noise to each of the three parameters for each simulated agent (from a standard normal distribution multiplied

by 0.05) to improve our coverage of possible parameter values. After generating the simulated behavior, we refitted the simulated behavior using fmincon in MATLAB (2019, The MathWorks). We used the best fit from 10 random starting configurations to avoid local minima. The correlations between the true simulated and fitted parameter values were: $K_{self=}$0.98; K_{other}=0.98; β=0.80. Thus, parameter recovery was reliable for all parameters.

Model identifiability

Data were simulated from 100 synthetic participants with each of our 24 models with parameter values drawn randomly from the ranges used for parameter recovery. For example, simulated data from the $2K1\beta$ used two randomly generated K parameters and one β parameter. We then fit the models to these data in the same way as the participant data and repeated the simulation and fitting process ten times for all 40 models. On each of the ten rounds, we designated the winning model as the one with the lowest total BIC across participants and also calculated the percentage of participants for which each model had the lowest BIC. Strong model identifiability is show by models where simulated data wins most often (summed across the ten rounds) and is best for a large percentage of participants (averaged across the ten rounds). Our winning model (model 7) was strongly identifiable, but models that contained a parameter on reward only or both effort and reward were in general not identifiable, and not considered further (Figures S3A and S3B).

Imaging methods

Scanning was conducted in a Siemens Prisma 3-Tesla MRI scanner to acquire T2*-weighted echo planar imaging (EPI) volumes with a BOLD contrast BOLD. EPI volumes were acquired at a 30 degree ascending oblique angle to the AC-PC line. The angle chosen decreased the impact of susceptibility artefacts in the orbitofrontal cortex, a method validated in previous studies.[103] Acquisition parameters were as follows: voxel size 2x2x2, 1mm gap; TE = 30ms; repetition time = 1254ms; flip angle = 90°; field of view = 2.16mm. A magnetization prepared rapid gradient echo (MPRAGE) sequence with 192 slices was used to obtain the structural scan (slice thickness = 1mm; TR = 1900ms; TE = 3.97ms; field of view = 192x192mm; voxel size = 1×1x1mm resolution).

Imaging pre-processing and analyses

Data were pre-processed and analyzed using SPM12 (Wellcome Department of Imaging Neuroscience, Institute of Neurology) and a standard pre-processing pipeline. Images were realigned and unwarped using a fieldmap and co-registered to the participant's own anatomical image. The anatomical image was processed using a unified segmentation procedure combining segmentation, bias correction, and spatial normalization to the MNI template using the New Segment procedure.[104] The same normalization parameters were then used to normalize the EPI images, which were then spatially smoothed using an isotropic Gaussian kernel at 8mm full-width at half-maximum.

Imaging design: multivariate analysis

Representational similarity analysis (RSA) of fMRI data was performed using SPM12, the RSA toolbox and custom scripts.[56] We estimated voxel activity patterns time-locked to the offer cue for each effort, reward and recipient combination by creating 50 columns in our GLM that corresponded to these combinations. In addition, the same GLM modelled the onset of force exertion and the onset of the outcome on self and other trials as separate regressors, the break periods, as well as 6 motion regressors. GLMs were inspected to ensure all events could be estimated independently from one another with minimal correlations (Figure S6). Due to the variability between participants in the number of repetitions of effort levels – which depended on participant choice behavior – a multivariate analysis was not suitable for the force period (since the difference in number of repetitions could impose structure on the RDMs).

Both ROI analyses and whole-brain searchlight analyses were based on smoothed data.[105] We applied multivariate noise normalization to the voxel activity patterns to improve reliability[56,70] and calculated the correlation distance using the pdist function in Matlab. The correlation distance metric was chosen as a measure that is magnitude insensitive to the BOLD signal, and thus makes separate predictions from the univariate trial-by-trial model-based analysis or by using alternative distance metrics such as Euclidean distance.[60,70] For the ROI analysis, anatomical masks were realigned to be in the same voxel space as participant scans and then custom scripts were used to calculate the resulting representational dissimilarity matrices in a particular ROI with regression coefficients that were spatially pre-whitened.

As in previous RSA studies,[56,106–108] the diameter of the searchlight sphere was 15mm (approximately 100 voxels) and we used the group level mask to define the volume for the searchlight analysis. We note that the ROI analysis and whole brain searchlight analyses are not directly comparable, but instead complement one another.[56,106] The shape of the searchlight sphere is insensitive to the precise anatomical boundaries of a particular ROI. In addition, hypothesis driven and anatomically defined ROIs can capture pattern information that may not be visible in a searchlight. For example, the ACCg ROI is more sensitive to subtle pattern information as it contains 5 times more voxels than the searchlight sphere. The brain searchlight maps were correlated with each model RDM using Kendall's τ_A to parallel the ROI analysis. The searchlight was also performed using adapted scripts from the RSA toolbox (Varazzani et al.,[10] original surface-based searchlight scripts from by Joern Diedrichsen and Naveed Ejaz, code available at https://github.com/rsagroup/rsatoolbox). The searchlight definition was executed using Freesurfer's reconall command and depended on cortical reconstruction and alignment.[11–13] This procedure incorporated subject-specific anatomy by defining cortical searchlights on the 2D surface. As in the ROI analysis, regression coefficients were spatially pre-whitened within the searchlight using the RSA toolbox.

Formal conjunction analyses were run to determine the areas that responded across self/other recipient conditions. Comparison analyses between self and other conditions ([-1 1] for other > self or [1 -1] for self > other) were also run at each second-level design to reveal the areas that responded specifically to one condition, yielding areas of domain-specific activation. For ROI analysis we tested whether correlations were significantly different from zero using non-parametric one-sided Wilcoxon signed-rank tests across participants. One-sided tests are used when comparing model RDMs and brain RDMs to one another. This is because only positive correlations are theoretically plausible. If two RDMs have a negative correlation, they would not be concordant as theoretically the model would be predicting that the largest distances in the data are smallest. Thus, a negative correlation can only happen under H0.[56] We also tested whether correlations for self were significantly different from correlations for other with either self or other representations could have provided a better explanation of the brain RDMs. All reported comparisons survived FDR correction at $p < 0.05$.[56] For one-sided tests this was across 24 comparisons (4 brain RDMs, 3 model RDMs, 2 recipients) and for two-sided tests between recipients, 12 comparisons (4 brain RDMs, 3 model RDMs). ROIs were constructed using anatomical masks from regions of strong a priori interest and that could distinguish dACC from more ventral portions of ACC in the gyrus. These ROIs were thus the dmPFC/dACC (4088 voxels),[18] anterior insula (2429 voxels),[18] ACCg (525 voxels)[36,37,39,41,62] and TPJ (1996 voxels)[46,49] (see https://osf.io/tm45q for mask.img and.nii files). As outlined in the introduction, the ACCg has been repeatedly highlighted as a core neural region for processing social information with theoretical and empirical accounts predicting that this region is critical for motivating prosocial effort.[6,36,37,72,71] In contrast, the dACC/dmPFC and anterior insula (AI) have previously been linked to coding the subjective value of choosing to work vs. rest in contexts of both physical and cognitive effort,[18] suggesting a domain general response. Finally, the TPJ has often been implicated in social cognition and prosocial behavior, and encodes effort costs differently when behaviors switch from being cooperative to competitive.[45–49] At the request of reviewers, two further exploratory ROIs were included in ventral striatum and vmPFC (for full results see supplemental results, Figure S5 and Table S3 and S4). In addition to these four ROIs, we conducted exploratory whole-brain analyses for completeness and considered areas significant that survived correction for multiple comparisons at the cluster level ($p < 0.05$, corrected for family-wise error (FWE) after thresholding at $p < 0.001$[109]), both in the data-driven searchlight and in the univariate analyses.

Note we took this analytical approach of evaluation brain RDMs in separate 25 x 25 matrices for self and other trials rather than calculating a full 50x50 matrix representing all conditions in the same model (e.g. Hall-McMaster et al.,[110]). Self and other brain RDMs allowed us to directly test whether a brain area represents information on self or other trials significantly (or not), and whether it does so significantly differently between self and other trials, which is crucial for answering questions about the 'specialization' of signals.[12]

Imaging design: Univariate analysis

Three event types were used to construct regressors which would be convolved with Statistical Parametric Mapping's canonical hemodynamic response function.[111] As in the multivariate analysis, onsets were modelled using regressors for the choice phase, force phase, and outcome phase. Each regressor was associated with a parametric modulator. The choice phase regressor was associated with the parametric modulator of the subjective value difference of the chosen option, to parallel previous work,[18] force with an effort required parametric modulator (0 if no effort or if chosen the level of effort on offer), and outcome with an outcome parametric modulator (the reward outcome received on each trial). Each parametric modulator was separated by recipient (self or other). The resulting GLM had 12 columns: the first four represented self and other choices as well as their subjective values (SVs), the next four represented the self and other force exerted as well as their force (effort) parametric modulators, and the final four held self and other outcomes and their parametric modulators. Additional regressors modelled the break phase and missed trials in participants who had missed trials. First-level design matrices were inspected to ensure the different parametric modulators could be estimated with independence. Crucially, the choice phase and effort phases were decorrelated by introducing jittering and ensuring that the resulting parametric modulators were uncorrelated in the design (maximum correlation = 0.01, Figure S6A).

First-level contrast images built from the above-described design matrix focused on self and other modulators of choice SV and force. These images were then inputted into two second-level flexible-factorial designs that tested for neural regions that tracked the predicted SV during the choice period and the level of effort during the force period. Conjunction analyses were run to determine the areas that responded across self/other recipient conditions. Comparison analyses between self and other conditions (-1 1 for other > self or 1 -1 for self > other) were also run at each second-level design to reveal the areas that responded specifically to one condition, yielding areas of domain-specific activation. Analyses were reported at $p < 0.05$, family-wise error (FWE) corrected at the cluster level after thresholding at $p < 0.001$ across the whole brain or at $p < 0.05$ small-volume corrected at the peak voxel level, using anatomical masks from regions of strong a priori interest, the dmPFC/dACC,[18] anterior insula,[18] ACCg[36,37,39,41,62] and TPJ.[46,49] In addition to correcting for family-wise error (FWE) small volume-correction in independent anatomically defined regions of interest, we performed non-parametric permutation tests to determine the cluster level for FWE correction at $p < 0.05$ after thresholding at $p < 0.001$ (10,000 Monte-Carlo simulations).[112,113,114] All reported clusters within the small volumes exceeded this threshold, supporting the validity of the FWE multiple-comparison correction procedure.

Exploratory ROIs in vmPFC and VS

We ran an additional exploratory RSA analysis including vmPFC (areas 11m and 14m) and also ventral striatum (Harvard-Oxford Atlas) at the request of reviewers. These regions did not form our a priori ROIs, which were based on existing literature and meta-analyses,[16] so we interpret these results with some caution. We also note that in fMRI dropout in vmPFC/OFC is very common

and thus other approaches such as non-human work or lesion approaches might be better suited to addressing the role of vmPFC.

Intriguingly we found that vmPFC carried multivariate representations of reward and subjective value for self, but only subjective value for other (Figure S5; Table S4 and S5). This suggests that vmPFC may represent domain general subjective value signals for self and other but preferentially represents rewards for self. This fits with prior work suggesting reward is encoded preferentially for self in vmPFC[17,18] and extends this to show multiple signals represented by vmPFC in different contexts. We also observed stronger representations of reward than effort in vmPFC for self (Table S5).

Connectivity analysis

We ran two further analyses to address the connectivity profile of ACCg. First we performed a 'representational connectivity analysis' whereby we correlated the ACCg other brain RDM with the dACC/dmPFC and AI brain RDMs on other trials and compared them to the same brain RDMs on self trials. This analysis revealed that ACCg representations on other trials correlated with dACC/dmPFC representations on other trials (mean rank correlation $\tau_A \pm SE = 0.31 \pm 0.01$, $p < 0.001$), as well as AI representations on other trials (mean rank correlation $\tau_A \pm SE = 0.32 \pm 0.01$, $p < 0.001$). We next examined whether this connectivity was stronger than connectivity with representations in dACC/dmPFC on self trials, which was indeed the case (self mean rank correlation $\tau_A \pm SE = 0.02 \pm 0.01$; Wilcoxon two-sided signed rank test Z=-6.85, effect size *r*=0.87 [0.87, 0.87], $p < 0.001$). The same pattern of stronger connectivity on other than self trials was also evidence between ACCg and AI (self mean rank correlation $\tau_A \pm SE = 0.03 \pm 0.01$; Wilcoxon two-sided signed rank test Z=-6.85, effect size *r*=0.87 [0.87, 0.87], $p < 0.001$). Thus ACCg representations for other correlated with representations in regions that we identified as signalling domain general subjective value.

We also conducted a PPI analysis on the univariate ACCg signals during the force period that scaled with effort for other more strongly than for self. We identified a seed region in ACCg (2mm sphere) and examined both positive and negative connectivity between this area and the whole brain. The univariate analysis did not reveal any functional connectivity with other regions that survived whole brain correction or small volume correction in any of our ROIs.

Current Biology, Volume *32*

Supplemental Information

Distinct neural representations

for prosocial and self-benefiting effort

Patricia L. Lockwood, Marco K. Wittmann, Hamed Nili, Mona Matsumoto-Ryan, Ayat Abdurahman, Jo Cutler, Masud Husain, and Matthew A.J. Apps

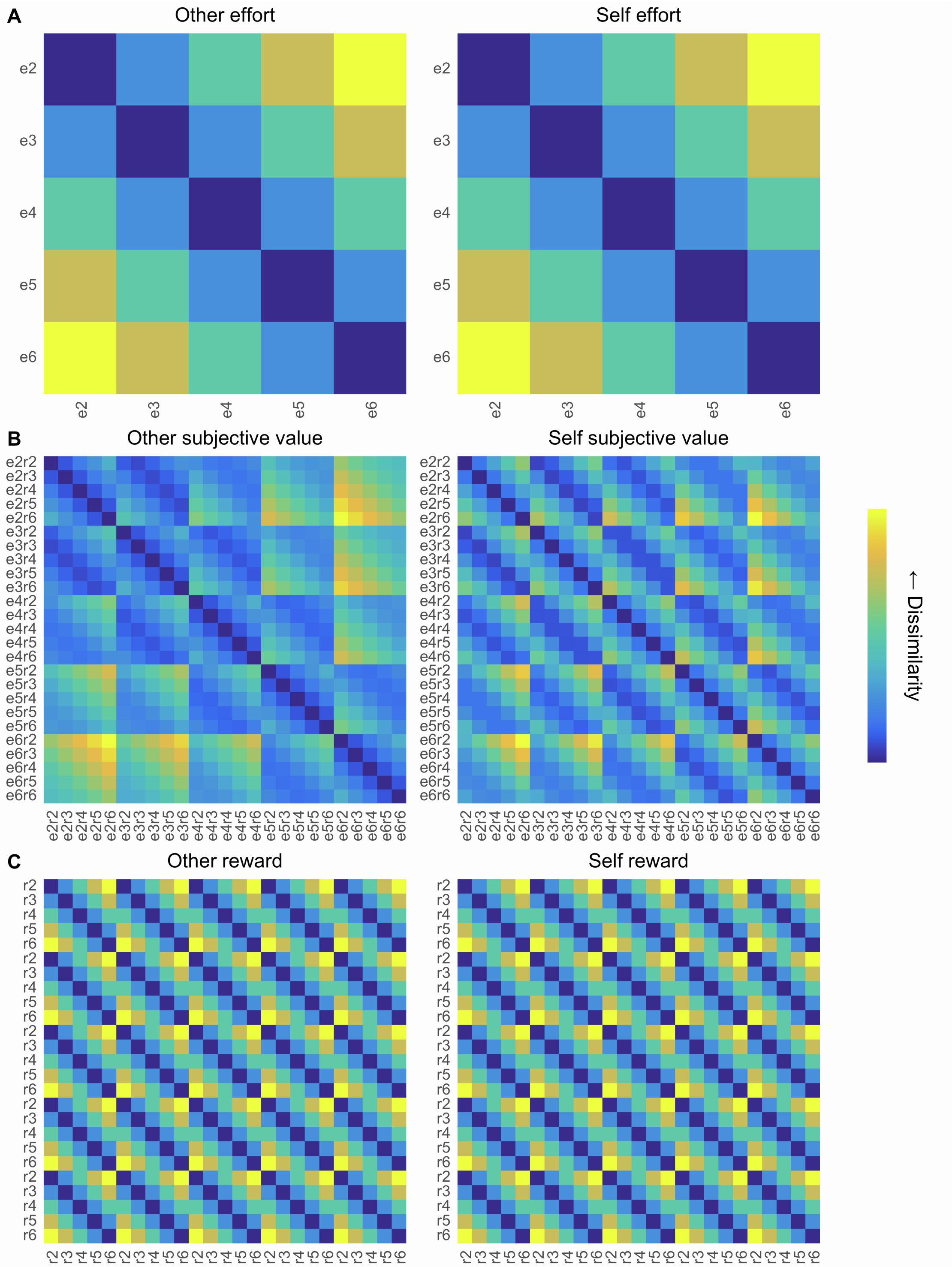

Figure S1. Model RDMs for effort, subjective value and reward. Related to Figure 1. Six 25 x 25 (5 effort and 5 reward levels) model representational dissimilarity matrices (RDMs) were constructed at the offer stage to analyse activity during effort-based decisions separately for the self and other conditions. These coded for different task features in multivariate space. **(A)** For the effort model RDMs, this was the Euclidean distance between effort levels on offer. **(B)** For the subjective value RDMs, this was the Euclidean distance between subjective values of offers based on the winning computational model with separate discount parameters (*K*) for self and other trials. Each participant's individual *K*

parameter was used. **(C)** For the reward RDMs this was the Euclidean distance between reward levels on offer. Yellow colours show conditions are more dissimilar whereas dark blue colours show conditions are more similar in terms of the Euclidean distance between conditions.

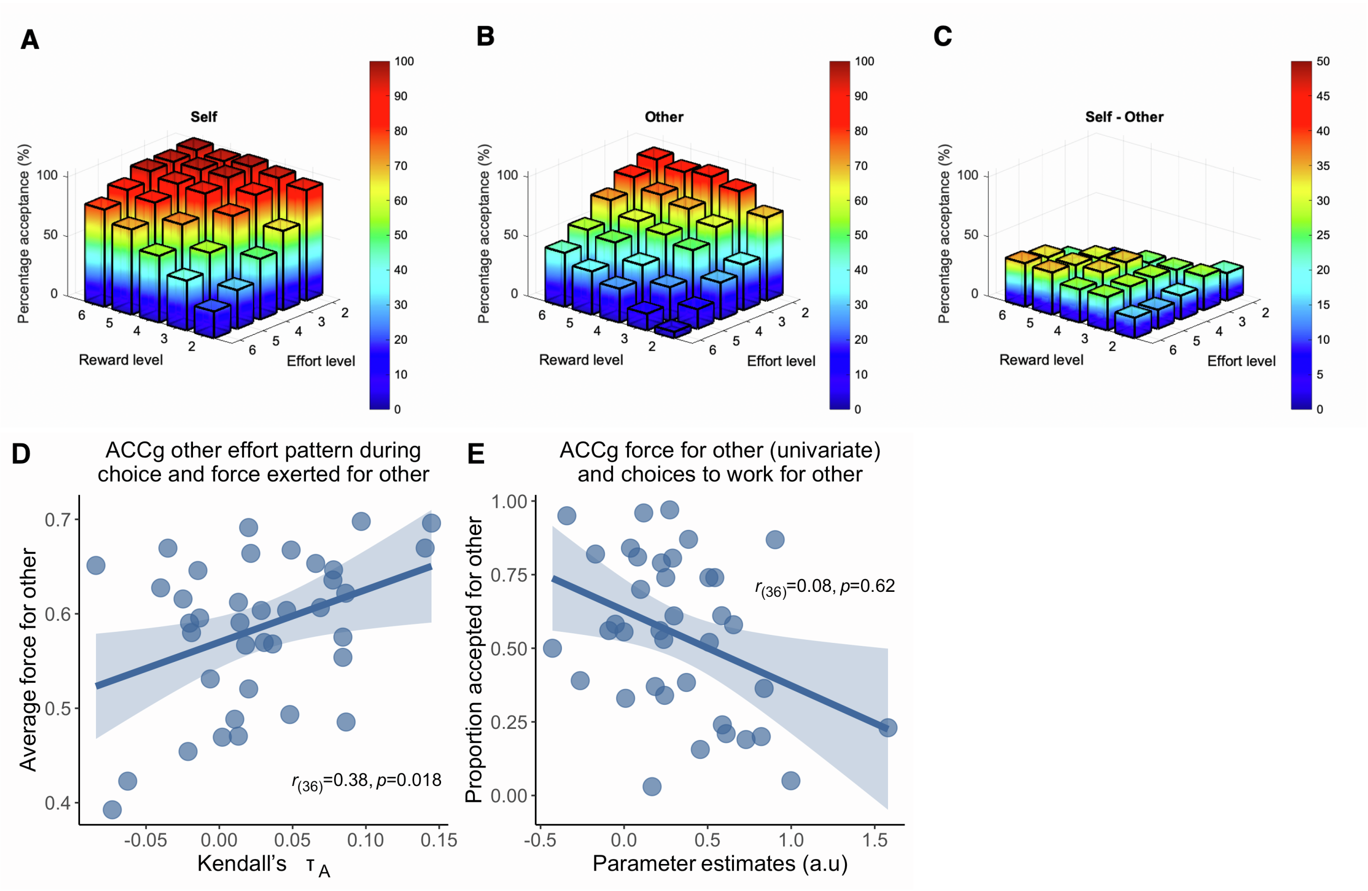

Figure S2. Supplemental behavioural analysis of choice and force. Related to Figure 2. 3D plots showing percentage acceptance of work over rest offers on self and other trials and the difference between them**. (A)** Percentage acceptance on self trials as a function of reward and effort level. **(B)** Percentage acceptance on other trials as a function of reward and effort level. **(C)** Difference in percentage acceptance between self and other trials. **(D)** ACCg representations of effort for another person during the choice phase, taken from the RSA analysis, positively correlate with amount of force subsequently exerted for other (Pearson's $r_{(36)}$=0.38, p=0.018). **(E)** Univariate responses in ACCg to force required for other negatively correlate with proportion of choices to benefit other (Pearson's $r_{(36)}$=-0.38, p=0.018).

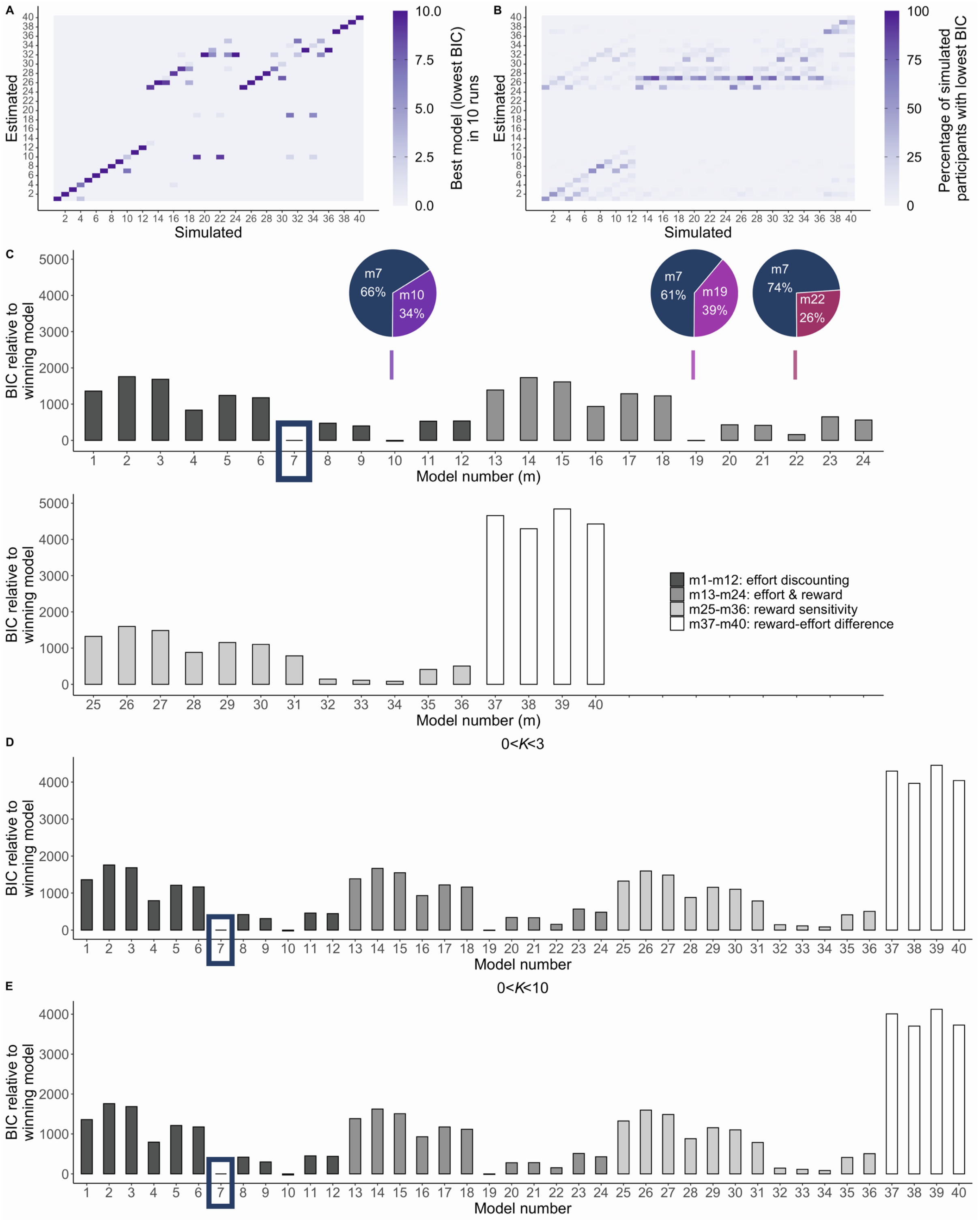

Figure S3. Model identifiability and model comparison with all models and different *K* bounds. Related to Figure 2. (A) Data simulated from each of the 40 models (see section 'Computational Modelling' below for details) for 100 participants. The model comparison procedure identifies the model that simulated the data, demonstrated by the strong diagonal, for models 1-12, which include an effort discounting and an inverse temperature parameter. However, models 13-24 (additional reward sensitivity parameter), models 25-36 (only a reward sensitivity parameter, no effort discounting parameter) and models 37-40 (linear models only, parameter scaling difference between effort and reward) show poor identifiability. **(B)** We repeated the simulations and fittings ten times and quantified the winning model as

with the modelling of participants' data as the model with the lowest Bayesian Information Criterion (BIC), summing the number of times that model won across the ten runs. We also calculated the percentage of simulated participants for which each model had the best fit to the data and averaged this over the ten runs. **(C)** Model comparison of all 40 models shows that even those models with comparable BICs explain behaviour in fewer participants than model 7. **(D)** We also tested empirically whether the estimated *K's* were sensitive to the bounds we placed on them. Increasing the maximum *K* value to 3 did not change the winning model (Model 7). **(E)** Repeating the model fitting with the upper *K* bound as 10 also gave identical results.

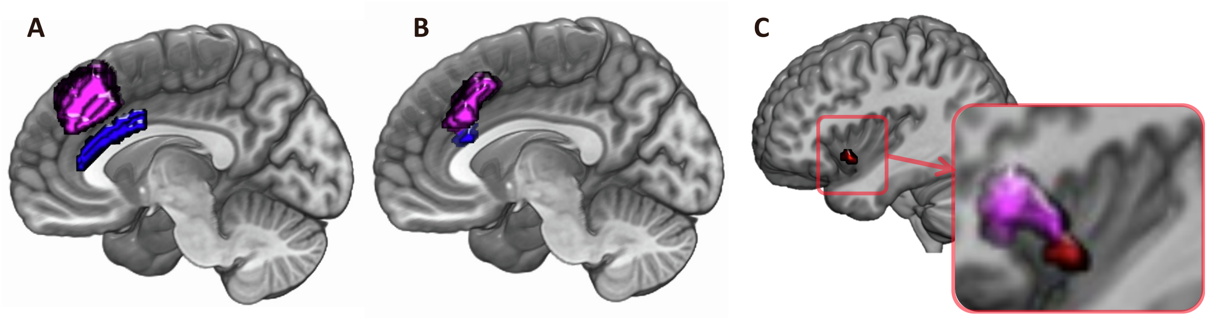

Figure S4. Distinct portions of the cingulate cortex involved in domain general and domain specific computations. Related to Figure 4, Table S6 and S7. (A) anatomical regions of interest in ACCg (blue) and dACC/dmPFC (pink) overlaid on an anatomical scans of the medial surface. Notably, the two areas of the cingulate cortex are distinct. Whereas the ACCg signalled representations of effort for others only, the dACC/dmPFC signalled patterns of subjective value for both self and other. **(B)** univariate analyses show that activation in the ACCg (blue) for force exerted for others only is distinct from a separate areas of the anterior cingulate cortex in the dACC/dmPFC (pink) that negatively tracked trial-by-trial subjective value for both self and other. **(C)** univariate analysis show that activation in the ventral anterior insula (vAI) that responds more strongly on self than other trials does not overlap with the domain general portion of anterior insula (pink) that tracks subjective value in a conjunction analysis for both self and other.

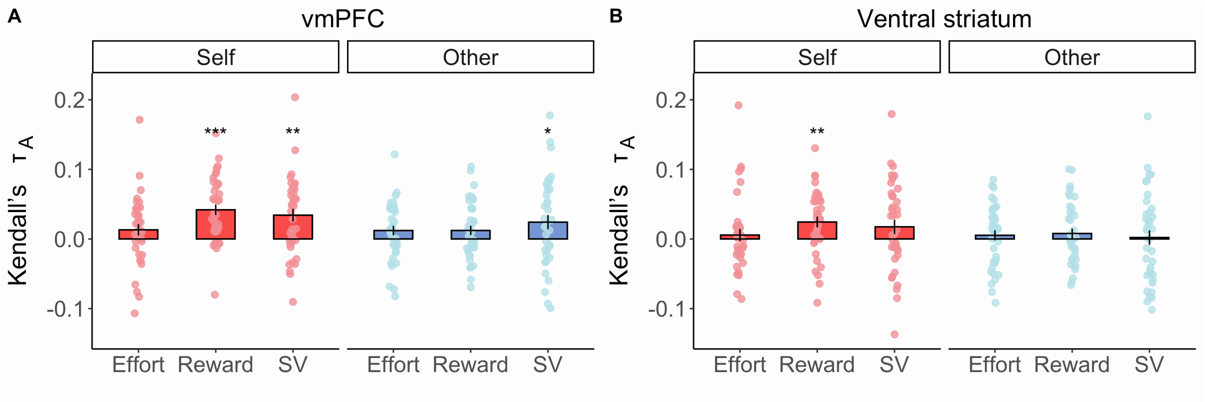

Figure S5. vmPFC and VS multivariate patterns of effort, reward and subjective value. Related to Table S6 and STAR Methods. We conducted additional exploratory analysis with two ROIs in vmPFC and VS that have more recently been suggested to encode effort, reward and subjective value. We found vmPFC coded reward and subjective value for self but only subjective value for other. In contrast ventral striatum only encoded reward for self. Reward was represented more strongly than effort in vmPFC for self but this was not the case for other (Table S6).

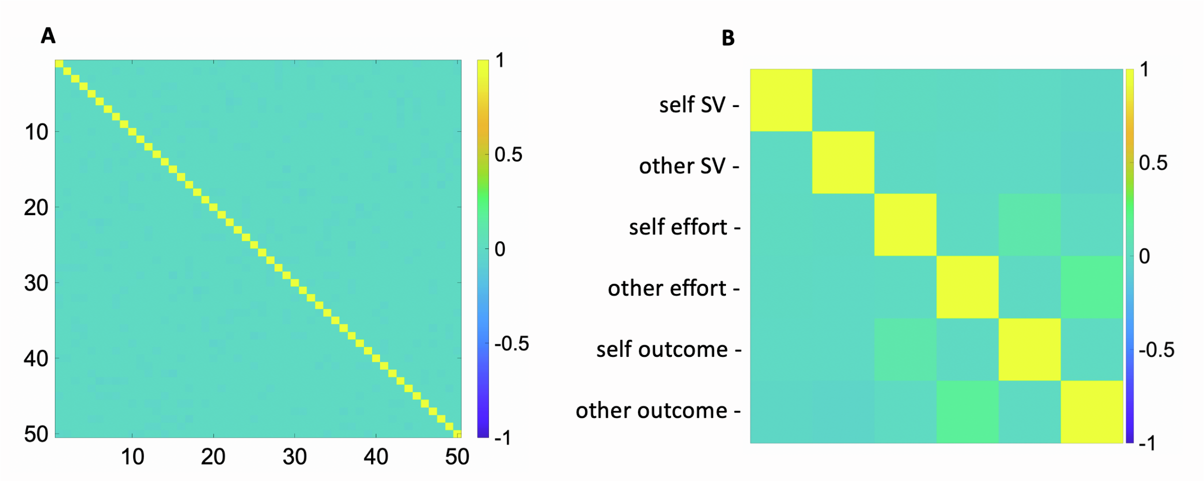

Figure S6. Multivariate and univariate GLMs were decorrelated. Related to STAR Methods. (A) Correlation between regressors in the Representational Similarity Analysis GLM. A 50x50 GLM of regressors modelled each effort/reward combination separately for self (columns 1-25) and other (columns 26-50) trials. Within this GLM all correlations were below r<l0.007l, indicating that conditions could be appropriately estimated with independence from one another. The GLM also modelled the onset of the force period and the onset of the outcome for each recipient (self and other). **(B)** Correlation between regressors in the univariate GLM. The correlations between choice (subjective value) and effort parametric regressors were below r<l0.01l, indicating that all conditions of interest (subjective value at the offer stage, effort required at the force stage) could be appropriately estimated with independence from one another. SV: subjective value.

Table S1. Generalised linear mixed-effects model predicting choices. Related to Figure 2.

	OR	SE	CI low	CI up	Z	χ2	df	*p*
(Intercept)	6.98	2.52	3.44	14.15	5.39			
Recipient (Self vs. Other)	4.04	0.99	2.49	6.54	5.66	23.05	1	0.001
Effort	0.13	0.02	0.09	0.17	-12.38	64.24	1	0.001
Reward	6.10	1.18	4.18	8.90	9.38	46.05	1	0.001
Recipient (Self vs. Other) * Effort	1.20	0.09	1.03	1.40	2.40	6.53	1	0.011
Recipient (Self vs. Other) * Reward	1.31	0.11	1.11	1.55	3.20	9.51	1	0.003
Effort * Reward	1.18	0.11	0.99	1.42	1.83	3.38	1	0.088
Recipient (Self vs. Other) * Effort * Reward	0.97	0.05	0.87	1.09	-0.47	0.19	1	0.67

Note. OR: odds ratio, SE: standard error, CI: 95% confidence interval for odds ratio, low: lower CI, up: upper CI, *p* values are from type 2 tests of fixed effects using parametric bootstrapping (see Methods).

Table S2. Linear mixed-effects model predicting normalised force. Related to Figure 3.

	$\chi 2$	df	*p*
Recipient (Self vs. Other)	7.09	1	0.013
Effort	409.15	4	0.001
Reward	65.66	4	0.001
Recipient (Self vs. Other) * Effort	5.36	4	0.24
Recipient (Self vs. Other) * Reward	13.21	4	0.014
Effort * Reward	49.88	16	0.001
Recipient (Self vs. Other) * Effort * Reward	42.03	16	0.002

Note. df: degrees of freedom, *p* values are from type 2 tests of fixed effects using parametric bootstrapping (see Methods).

Table S3. Kendall's τ_A correlations between brain RDMs and model RDMs for additional ROIs. Related to STAR Methods.

		Mean	SE	*p*	FDR *p*
Other effort RDM	vmPFC	0.01	0.01	0.03	0.06
	VS	0.01	0.01	0.24	0.29
Self effort RDM	vmPFC	0.01	0.01	0.03	0.06
	VS	0.01	0.01	0.61	0.61
Other reward RDM	vmPFC	0.01	0.01	0.05	0.09
	VS	0.01	0.01	0.24	0.29
Self reward RDM	vmPFC	0.04	0.01	<0.001	<0.001
	VS	0.02	0.01	0.00	0.01
Other subjective value RDM	vmPFC	0.02	0.01	0.02	0.05
	VS	0.00	0.01	0.49	0.54
Self subjective value RDM	vmPFC	0.03	0.01	<0.001	0.00
	VS	0.02	0.01	0.06	0.09

Note. RDM: representational dissimilarity matrix, Mean: Kendall's τ_A correlations between brain RDMs and model RDMs, SE: standard error of the mean, FDR *p*: false-discovery rate corrected *p* value across 12 comparisons.

Table S4. Comparing RDMs between effort and reward for each recipient. Related to STAR Methods.

		Z	*r*	CI low	CI up	*p*
Reward vs. effort self RDM	vmPFC	-2.28	0.37	0.06	0.62	0.02
	VS	-1.49	0.24	0.02	0.54	0.14
Reward vs. effort other RDM	vmPFC	-0.50	0.08	0.01	0.41	0.62
	VS	-0.17	0.03	0.00	0.37	0.86

Note. RDM: representational dissimilarity matrix, *r*: standardised effect size, CI: 95% confidence interval for odds ratio, low: lower CI, up: upper CI, values are from Wilcoxon two-sided rank tests comparing effort and reward.

Table S5. Kendall's τ_A correlations between brain RDMs and model RDMs. Related to Figure 4.

		Mean	SE	*p*	FDR *p*
Other effort RDM	ACCg	0.026	0.009	0.005	0.009
	AI	0.021	0.008	0.006	0.009
	dACC/dmPFC	0.029	0.008	0.001	0.003
	TPJ	0.033	0.010	0.001	0.003
Self effort RDM	ACCg	0.002	0.009	0.610	0.610
	AI	0.008	0.009	0.400	0.420
	dACC/dmPFC	0.016	0.012	0.160	0.170
	TPJ	0.024	0.013	0.026	0.032
Other reward RDM	ACCg	0.009	0.007	0.16	0.170
	AI	0.020	0.008	0.006	0.009
	dACC/dmPFC	0.025	0.007	0.001	0.003
	TPJ	0.016	0.007	0.027	0.033
Self reward RDM	ACCg	0.038	0.008	<0.001	<0.001
	AI	0.035	0.009	<0.001	0.002
	dACC/dmPFC	0.041	0.009	<0.001	<0.001
	TPJ	0.026	0.009	0.005	0.008
Other subjective value RDM	ACCg	0.038	0.014	0.009	0.013
	AI	0.051	0.013	<0.001	<0.001
	dACC/dmPFC	0.073	0.011	<0.001	<0.001
	TPJ	0.044	0.012	<0.001	<0.001
Self subjective value RDM	ACCg	0.055	0.012	<0.001	<0.001
	AI	0.047	0.012	<0.001	<0.001
	dACC/dmPFC	0.064	0.012	<0.001	<0.001
	TPJ	0.026	0.011	0.018	0.024

Note. RDM: representational dissimilarity matrix, Mean: Kendall's τ_A correlations between brain RDMs and model RDMs, SE: standard error of the mean, FDR *p*: false-discovery rate corrected *p* value across 24 comparisons.

Table S6. Whole-brain RSA searchlight results. Related to Figure 5 and Figure 6.

Brain Region	L/R	Peak voxel			k	t	z
**Conjunction: RSA Effort**							
Precuneus	R	24	-70	42	1359	5.55	5.06
	R	38	-76	14		4.96	4.60
	R	28	-72	54		4.26	4.02
Precuneus	L	-18	-74	50	409	4.55	4.26
	L	-24	-64	58		3.92	3.73
	L	-24	-74	42		3.85	3.66
**Other > Self: RSA effort**							
No suprathreshold voxels							
**Self > Other: RSA effort**							
Postcentral gyrus	R	18	-46	66	340	4.84	4.49
	R	6	-44	68		4.06	3.85
	R	12	-58	64		3.72	3.55
**Conjunction: RSA Subjective value**							
Precentral gyrus	L	-38	-22	56	5941	4.85	4.50
ext. suppl. motor area	L	-10	-6	62		4.83	4.49
	L	-10	6	58		4.80	4.46
Superior frontal gyrus	L	-22	36	32	731	4.80	4.46
	L	-22	46	30		4.29	4.04
ext. dorsomedial prefrontal cortex	L	-20	40	24		4.27	4.03
Anterior insula	L	-46	18	6	563	4.11	3.89
ext. Inferior frontal gyrus	L	-56	16	22		4.10	3.88
	L	-32	30	10		3.99	3.79
Inferior parietal lobe	L	-48	-46	50	278	4.02	3.81
ext. superior parietal lobe	L	-32	-56	56		3.93	3.74
	L	-40	-58	56		3.79	3.61
**Other > Self: RSA subjective value**							
No suprathreshold voxels							
**Self> Other: RSA subjective value**							
Posterior cingulate	R	20	-20	50	578	4.78	4.45
	R	22	-24	42		4.76	4.43
ext. Posterior insula	R	32	-24	24		4.41	4.14
Midbrain/ventral tegmental area	R	4	-22	-16	291	4.44	4.16
	L	-2	-28	-18		4.28	4.04
	L	-10	-30	-28		3.75	3.57
**Conjunction: RSA Reward**							
Precentral gyrus	R	30	-14	56	1058	5.40	4.94
	R	30	-22	58		4.90	4.55
	R	34	-20	46		4.58	4.29
Cuneus	L	-12	-76	-22	2281	5.32	4.88
ext. lingual gyrus	L	-20	-82	-14		5.04	4.66
	L	-10	-86	-14		4.67	4.36
Precentral gyrus	L	-42	-14	60	1991	4.95	4.58
ext. inferior frontal gyrus	L	-38	18	30		4.42	4.15
ext. middle frontal gyrus	L	-38	4	52		4.39	4.12
Superior parietal lobe	L	-22	-66	58	319	4.45	4.18
ext. Inferior parietal lobe	L	-28	-52	48		3.61	3.46
Paracentral lobule	R	2	-42	58	285	4.30	4.05
ext. Precuneus	L	-6	-52	56		3.59	3.44
	L	-6	-38	52		3.52	3.38
**Other > Self: RSA reward**							
No suprathreshold voxels							
**Self > Other: RSA reward**							
Precuneus	R	34	-78	-40	312	4.59	4.29
	R	16	-68	-48		4.54	4.25
	R	26	-76	-42		4.32	4.07

Whole-brain searchlight results. For all regions, FWE P < 0.05 cluster-level whole-brain corrected after thresholding at p<.001. ext, extending into; k, cluster extent; L, left; PE, R, right.

Table S7. Whole-brain univariate results. Related to Figure 5 and Figure 6.

Brain Region	L/R	Peak voxel			k	t	z
**Conjunction: univariate subjective value**							
Dorsal anterior cingulate cortex	R	8	26	34	1359	5.16	4.75
	L	-6	16	46		5.14	4.74
	L	-6	28	34		4.70	4.38
**Other > Self: univariate subjective value**							
No suprathreshold voxels							
**Self > Other: univariate subjective value**							
No suprathreshold voxels							
**Conjunction: univariate force required**							
Precentral gyrus	L	-20	-28	64	116518	15.63	>8
ext. cerebellum	L	2	-70	-38		14.10	>8
	L	2	-60	-12		13.88	>8
**Other > Self: univariate force required**							
Posterior parietal lobe (temporo-parietal junction)	L	-50	-62	40	776	5.28	4.85
	L	-26	-68	56		3.57	3.42
Superior frontal gyrus (dorsomedial prefrotnal cortex)	L	-12	26	52	2133	4.91	4.55
	R	12	48	38		4.86	4.51
	L	-16	50	28		4.51	4.22
Superior temporal gyrus	L	-56	-38	-8	414	4.51	4.22
	L	-52	-46	-4		4.32	4.07
	L	-44	-34	-6		4.18	3.95

Whole-brain univariate results for parametric modulators of subjective value (at offer) and force required (at onset of effort). For all regions, FWE $P < 0.05$ cluster-level whole-brain corrected after thresholding at $p<.001$. ext, extending into; k, cluster extent; L, left; PE, R, right.
